# Neuroprotective actions of a fatty acid nitroalkene in Parkinson’s disease

**DOI:** 10.1038/s41531-023-00502-3

**Published:** 2023-04-07

**Authors:** Roberto Di Maio, Matthew T. Keeney, Veronika Cechova, Amanda Mortimer, Ahssan Sekandari, Pascal Rowart, J. Timothy Greenamyre, Bruce A. Freeman, Marco Fazzari

**Affiliations:** 1 Pittsburgh Institute for Neurodegenerative Diseases, Pittsburgh, PA 15213 USA; 2 Department of Neurology, University of Pittsburgh, Pittsburgh, PA 15213 USA; 3 Department of Pharmacology and Chemical Biology, University of Pittsburgh, 200 Lothrop Street, Pittsburgh, PA 15261 USA

**Keywords:** Cellular neuroscience, Target identification

## Abstract

To date there are no therapeutic strategies that limit the progression of Parkinson’s disease (PD). The mechanisms underlying PD-related nigrostriatal neurodegeneration remain incompletely understood, with multiple factors modulating the course of PD pathogenesis. This includes Nrf2-dependent gene expression, oxidative stress, α-synuclein pathology, mitochondrial dysfunction, and neuroinflammation. In vitro and sub-acute in vivo rotenone rat models of PD were used to evaluate the neuroprotective potential of a clinically-safe, multi-target metabolic and inflammatory modulator, the electrophilic fatty acid nitroalkene 10-nitro-oleic acid (10-NO_2_-OA). In N27-A dopaminergic cells and in the substantia nigra pars compacta of rats, 10-NO_2_-OA activated Nrf2-regulated gene expression and inhibited NOX2 and LRRK2 hyperactivation, oxidative stress, microglial activation, α-synuclein modification, and downstream mitochondrial import impairment. These data reveal broad neuroprotective actions of 10-NO_2_-OA in a sub-acute model of PD and motivate more chronic studies in rodents and primates.

## Introduction

Parkinson’s disease (PD) is an age-related neurodegenerative disorder affecting the central, peripheral and autonomic nervous systems. Arguably, the best characterized degeneration occurs in the dopaminergic neurons of the substantia nigra pars compacta (SNpc). PD is the fastest growing neurodegenerative disease worldwide^1^ affecting >1% of individuals ≥60 year old (7–10 million), with men having a higher incidence than women^2^. In the U.S. in 2017, >1 million people had a diagnosis of PD, and ~60,000 cases are diagnosed yearly, with an economic burden of $52 billion. Available therapies only alleviate PD symptomatology without targeting underlying pathogenic mechanisms. As such, they do not slow or stop the relentless progression of the disease. In the next two decades, the incidence of PD is projected to double^3^; thus there is an imperative and presently unmet need to identify novel therapeutic targets and disease-modifying drug strategies.

The characteristic degeneration of nigrostriatal dopaminergic (DA) neurons in the SNpc in familial and idiopathic PD has been associated with various pathogenic stimuli, including oxidative stress, accumulation of toxic forms of α-synuclein, mitochondrial dysfunction, and neuroinflammation^4–6^. Notably, large cohort clinical studies investigating the effects of (a) antioxidants such as vitamin C, carotenoids, tocopherol, and coenzyme Q_10_ in inhibiting oxidative stress and mitochondrial dysfunction and (b) nonsteroidal anti-inflammatory drugs (NSAIDs), did not prevent neuroinflammation and showed no association with a reduced risk for developing PD^7–10^.

Recent evidence reveals that the stimulation of antioxidant and cytoprotective responses upon activation of nuclear factor erythroid-derived 2-like-2 (Nrf2) induces neuroprotective effects in animal models of PD. Of note, small molecule electrophiles such as sulforaphane and dimethyl fumarate (DMF) activate Nrf2-dependent gene expression with significant therapeutic potential in PD^11–14^. Our recent data reveals additional novel PD-related pathogenic events and potential therapeutic targets. These findings revealed (i) enhanced activation of the most abundant NOX isoform in brain, NADPH oxidase isoform 2 (NOX2) increased the generation of ROS and lipid peroxidation products^15^, (ii) aberrant leucine-rich repeat kinase 2 (LRRK2) kinase activity that correlated with endo-lysosomal impairment^16,17^, and α-synuclein-mediated impairment of mitochondrial protein import with resultant mitochondrial dysfunction^18^.

Nitro-fatty acids (NO_2_-FA) are a class of nitric oxide (NO) and nitrite (NO_2_^−^)-derived endogenous fatty acid mediators that are detectable in humans, plants and insects^19–24^. The partial positive charge (δ^+^) on the alkenyl β-carbon adjoining the electron-withdrawing nitro group (NO_2_), confers NO_2_-FA with an electrophilic character that promotes a reversible Michael addition with nucleophilic cysteines (Cys). This results in the post-translational modification (PTM) of target proteins that have uniquely reactive Cys moieties^25,26^. Cytoprotective and anti-inflammatory responses are hallmarks of the signaling actions of nitro-oleic acid (NO_2_-OA), since different model systems and clinical studies show this small molecule electrophile: (a) activates Nrf2, PPAR-γ and heat shock protein-regulated gene expression, (b) inhibits NF-κB and stimulator of interferon-γ (STING) signaling, and (c) inhibits the catalytic activities of soluble epoxide hydrolase, cyclooxygenase-2, 5-lipoxygenase, and xanthine oxidoreductase^27–33^. Beneficial actions of NO_2_-OA have been reported in animal models of inflammatory diseases including inflammatory bowel disease, heart and kidney ischemia-reperfusion, adriamycin-induced renal dysfunction, sepsis, cutaneous inflammation, and obesity-induced hepatic steatosis^28,34–41^. These beneficial responses in cell and murine models motivated preclinical and clinical safety studies of a synthetic homolog nitro-oleic acid, the specific regioisomer 10-nitro-octadec-9-enoic acid (10-NO_2_-OA).

The impact of the signaling and pharmacological effects of any NO_2_-FA in PD have not been reported. Herein, we addressed this gap in knowledge by evaluating the therapeutic potential of 10-NO_2_-OA in both in vitro and in vivo models of PD. We report that 10-NO_2_-OA induces Nrf2-dependent anti-inflammatory and cytoprotective responses and limits critical PD-related pathogenic events, including oxidative stress, α-synuclein-dependent mitochondrial import impairment, NOX2 and LRRK2 hyperactivation and microglial activation.

## Results

### Cytotoxicity and neuroprotective actions of 10-NO_2_-OA in vitro

The N27-A rat dopaminergic neural cell model for PD^42^ is typically cultured in 10% fetal bovine serum (FBS). Since serum nucleophiles can undergo Michael addition with 10-NO_2_-OA and high concentrations of nitroalkenes can be toxic, we assessed cell viability of increasing 10-NO_2_-OA concentrations in 2, 5, and 10% FBS. Cells supplemented with 10% FBS showed the same cell viability as at 5 and 2% FBS (Supplementary Fig. 1a). As expected, cell viability in presence of elevated concentrations of 10-NO_2_-OA (5–10 µM) was limited by 2% but not 10% FBS. Lower concentrations of 10-NO_2_-OA (0.5–2.5 µM) in 2% FBS did not affect cell viability. No loss of cell viability was noted after treatment with 50 nM rotenone, 2.5 μM 10-NO_2_-OA, or rotenone + 10-NO_2_-OA, when compared with vehicle (Supplementary Fig. 1b). Further experiments with N27-A cells thus used 2% FBS.

Hyperactive NOX2 and LRRK2 kinase activities have been reported in dopaminergic neurons in post-mortem brain tissue of patients with idiopathic PD (iPD)^15,16^, with these activities being linked to the pathogenesis of PD. The potential neuroprotective actions of 10-NO_2_-OA on rotenone-induced NOX2 and LRRK2 activities were evaluated in N27-A cells. To assess NOX2 activation in situ we utilized a proximity ligation (PL) assay that detects the interaction between NOX2 (gp91^phox^) and its regulatory subunit p47^phox^ (PL p47^phox^:NOX2)^15^. Non-cytotoxic concentrations of 10-NO_2_-OA inhibited the rotenone-induced p47^phox^:NOX2 PL signal (Supplementary Fig. 2a). Consistent with decreased NOX2-derived, rotenone-induced cytoplasmic reactive oxygen species production, dihydroethidium (DHE) oxidation was significantly reduced by 10-NO_2_-OA (Supplementary Fig. 2a). 10-NO_2_-OA also inhibited LRRK2 hyperactivation in rotenone-treated N27-A, as measured by PL pS1292:LRRK2^16^ (Supplementary Fig. 2b). Notably, 10-NO_2_-OA activated Nrf2-regulated gene expression in N27-A cells. This was reflected by the increased expression of the Nrf2 target genes heme oxygenase-1 (HO-1, Hmox1), NAD(P)H dehydrogenase quinone-1 (Nqo-1), and glutamate–cysteine ligase modifier subunit (Gclm) (Supplementary Fig. 3). In aggregate, these data reveal that 10-NO_2_-OA can prevent oxidative damage and LRRK2 activation elicited by rotenone in vitro.

### 10-NO_2_-OA induces neuroprotection by modulating key PD pathogenic events in vivo

The neuroprotective signaling actions of 10-NO_2_-OA in vitro provided the rationale for exploring in vivo responses to orally-administered 10-NO_2_-OA in a sub-acute rotenone model of PD in rat^15,16,18^. The impact of 10-NO_2_-OA was measured with respect to Nrf2 pathway engagement and pathogenic events linked with PD, including oxidative stress, α-synuclein aggregation, NOX2 and LRRK2 hyperactivation, mitochondrial protein import impairment, and microglial activation.

### 10-NO_2_-OA activates Nrf2-dependent gene expression in SNpc

**Fig. 1:**
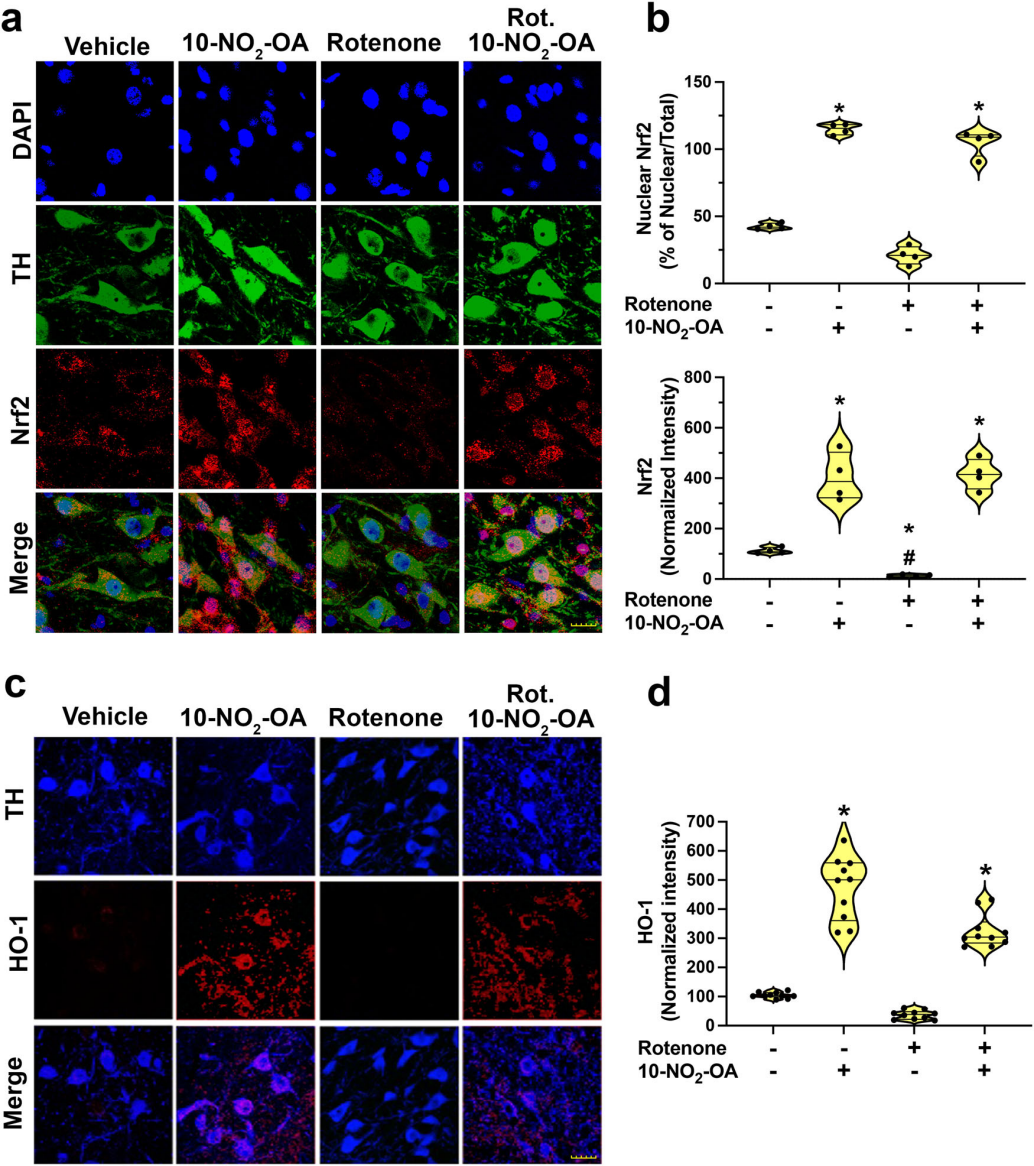
10-NO_2_-OA activates Nrf2 regulating heme oxygenase-1 expression in dopaminergic neurons of the SNpc. **a** Immunohistochemical assay for Nrf2 (Cohort 2, *n* = 4) reveals a significant nuclear recruitment of Nrf2 in DA neurons both in rats treated with 10-NO_2_-OA (45 mg/Kg) and Rotenone + 10-NO_2_-OA. A remarkable increase of Nfr2 expression in nigrostriatal DA neurons was also observed in 10-NO_2_-OA treatments, whereas rotenone induced downregulation of Nrf2 (Scale bar: 20 μm). **b** (upper graphic) Quantification of nuclear Nrf2. Symbols represent the percent of the ratio nuclear/total Nrf2 from a single animal (3 slices /brain). Statistical analysis was performed by one-way ANOVA with post hoc Bonferroni correction (*p < 0.0001 compared to Vehicle and Rotenone). **b** (lower graphic) Quantification of Nrf2 fluorescence intensity. Symbols represent the normalized means of the intensity (with Vehicle set at 100%) from a single rat (3 slices /brain). Statistical analysis was performed by one-way ANOVA with post hoc Bonferroni correction (*p < 0.0001 compared to Vehicle, #*p* < 0.0001 compared to 10-NO_2_-OA and Rotenone + 10-NO_2_-OA). **c** Immunohistochemical stain for HO-1 expression (Cohort 2). Rats treated with 10-NO_2_-OA (45 mg/Kg) or co-treated with 10-NO_2_-OA and rotenone displayed a significant increase of HO-1 protein expression (red) in dopaminergic nigrostriatal neurons (TH, blue—Scale bar: 35 μm). **d** Quantification of HO-1 fluorescence intensity. Symbols represent the normalized means of the intensity (with Vehicle set at 100) from a single rat (6 slices/brain). Statistical analysis was performed by one-way ANOVA with post hoc Bonferroni correction (*p < 0.0001 compared to Vehicle and Rotenone).

Since Nrf2 activation improves cellular redox homeostasis and induces cytoprotective responses, the targeting of Nrf2 signaling has recently shown therapeutic potential for PD^43^. Herein, we show significant 10-NO_2_-OA-dependent nuclear recruitment of Nrf2 in DA neurons detected by immunohistochemistry. Nuclear translocation of Nrf2 was also associated to a remarkable increase of Nfr2 expression in nigrostriatal DA neurons in 10-NO_2_-OA treatments, whereas rotenone induced downregulation of Nrf2 (Fig. 1a, b). Moreover, immunohistochemical quantitation showed 10-NO_2_-OA increased the expression of heme oxygenase-1 (HO-1), a hallmark of Nrf2 activation, in the substantia nigra pars compacta (SNpc) compared to both vehicle and rotenone-treated rats (Fig. 1c, d). 10-NO_2_-OA and its main non-electrophilic metabolite were quantified in rat brains by HPLC-MS/MS (Supplementary Fig. 4), affirming that 10-NO_2_-OA crosses the blood-brain-barrier (BBB) to engage Nrf2-dependent and other potential antioxidant and adaptive response mechanisms in the dopaminergic SNpc.

### 10-NO_2_-OA inhibits lipid peroxidation and 4-HNE modified α-synuclein formation

Lipid peroxidation is a response to inflammatory and metabolic-induced increases in reactive species generation. The fatty acid oxidation product 4-hydroxynonenal (4-HNE) was quantified immunohistochemically in SNpc. Rats treated with rotenone for 5 days displayed significant accumulation of 4-HNE in dopaminergic neurons, consistent with sustained oxidative stress. Rats administered increasing dose levels of 10-NO_2_-OA (5, 10, or 45 mg/Kg per day) showed decreased rotenone-induced 4-HNE accumulation (Supplementary Fig. 5).

**Fig. 2:**
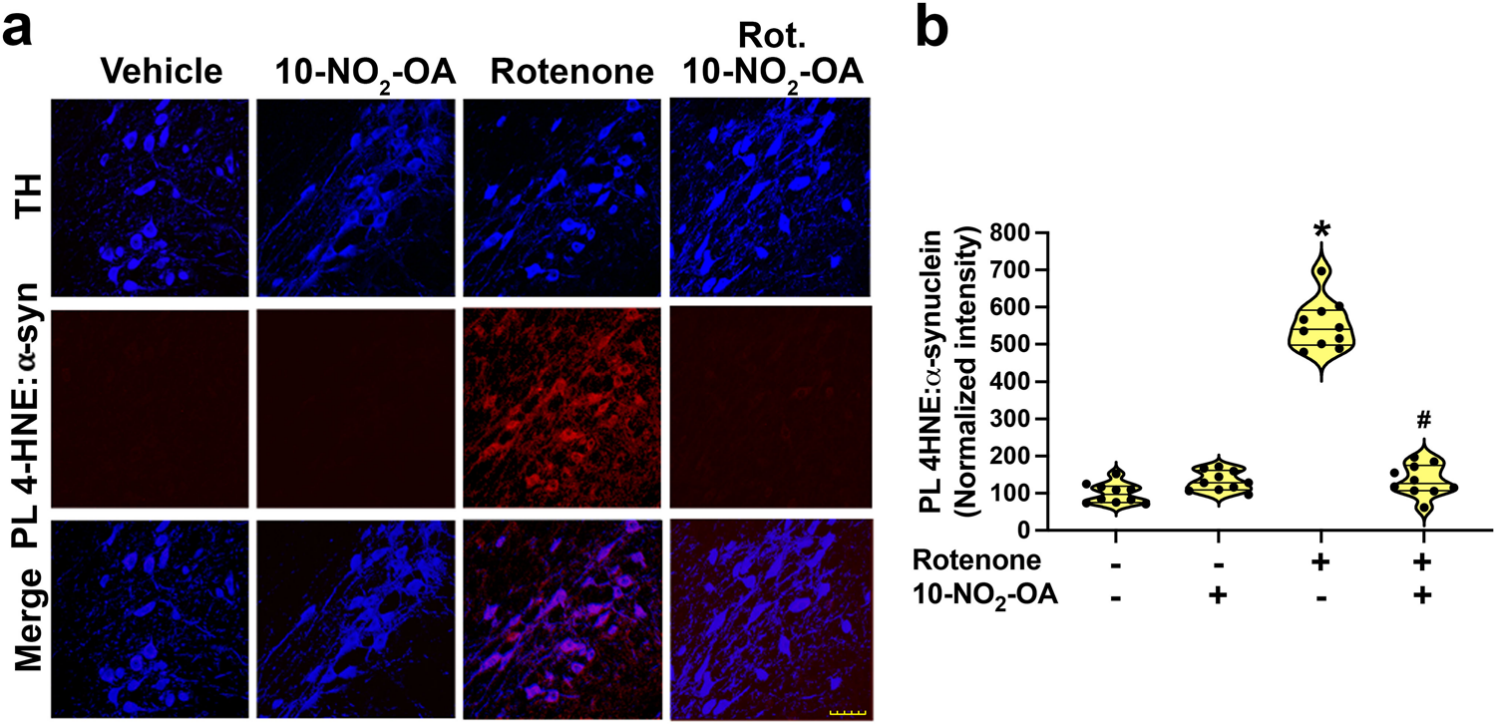
10-NO_2_-OA inhibits rotenone mediated accumulation of 4-HNE-α-syn adduct in the SNpc. **a** Confocal analysis of the detection of 4-HNE-α-synuclein adducts (PL 4-HNE α-synuclein) in SNpc DA neurons of rats (Cohort 2). Rotenone treatment caused a significant accumulation of 4-HNE-α-synuclein adducts (red) in nigrostriatal neurons (TH, blue) that was inhibited in rats treated with 10-NO_2_-OA (45 mg/Kg—Scale bar: 35 μm). **b** Quantification of PL 4-HNE-α-synuclein. Symbols represent the normalized means of the intensity (with Vehicle set at 100%) from a single rat (6 slices/brain). Statistical analysis was performed by one-way ANOVA with post hoc Bonferroni correction (*p < 0.0001 compared to Vehicle, ^#^*p* < 0.0001 compared to Rotenone).

The PTM of α-synuclein can have detrimental consequences to cellular health^44^. Oxidatively modified forms of α-synuclein that have accumulation of nitro-tyrosine or 4-HNE derivatives have a high propensity to aggregate and accelerate oligomerization^45^. The measurement of 4-HNE adducts of α-synuclein by proximity ligation assay (detected as PL 4-HNE-α-synuclein)^15^ showed that rats treated with 45 mg/Kg 10-NO_2_-OA displayed significant decreases in rotenone-induced 4-HNE-α-synuclein adduct accumulation (Fig. 2).

### 10-NO_2_-OA inhibits NOX2 activity

**Fig. 3:**
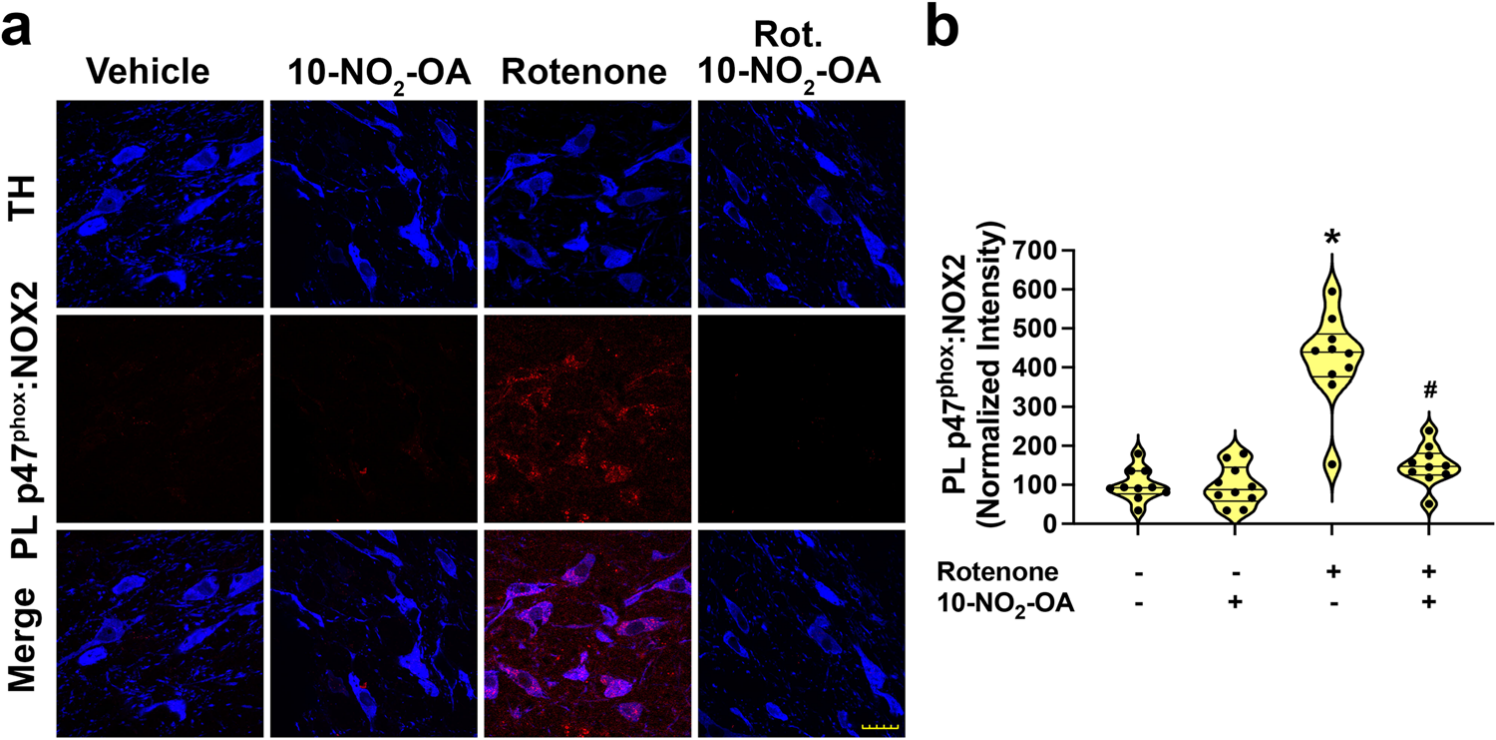
10-NO_2_-OA inhibits NOX2 activation in a sub-acute rotenone model of PD. **a** PL assay for in situ detection of NOX2 activation (PL p47^phox^:NOX2) (Cohort 2). Rats treated with 10-NO_2_-OA (45 mg/Kg) displayed inhibition of rotenone-induced NOX2 activation (red) in dopaminergic neurons of SNpc (TH, blue—Scale bar: 35 μm). **b** Quantification PL p47^phox^:NOX2 interaction. Symbols represent the normalized means of the intensity (with Vehicle set at 100%) from a single rat (6 slices/brain). Statistical analysis was performed by one-way ANOVA with post hoc Bonferroni correction (*p < 0.0001 compared to Vehicle, ^#^*p* < 0.0001 compared to Rotenone).

Neuronal NOX2 triggers an oxidative stress-related cascade of reactions of relevance to PD pathogenesis^15^. Herein, rats treated with rotenone for 5 days showed robust NOX2 activation in dopaminergic neurons of the SNpc. The administration of 10-NO_2_-OA significantly inhibited rotenone-induced NOX2 activation in a dose-dependent manner from 5 to 45 mg/Kg (Supplementary Fig. 6). Complete suppression of NOX2 activation by rotenone, equivalent to vehicle control levels, was observed in rats treated with 45 mg/Kg (Fig. 3 and Supplementary Fig. 6). Compared with vehicle-treated rats in the absence of rotenone, 45 mg/Kg 10-NO_2_-OA did not impact NOX2:PLA intensity.

### 10-NO_2_-OA prevents rotenone-induced LRRK2 activation

**Fig. 4:**
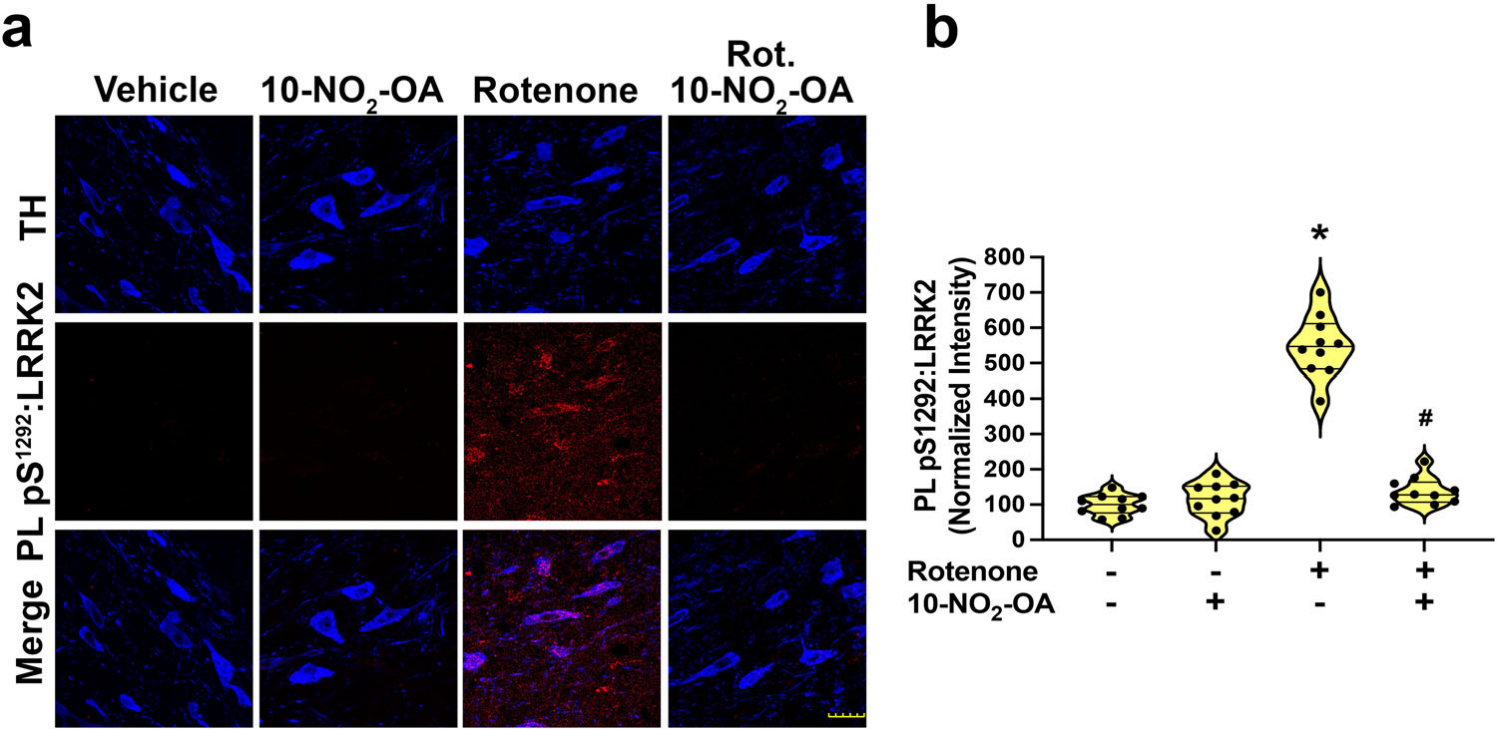
10-NO_2_-OA inhibits rotenone-induced LRRK2 activation in dopaminergic neurons of SNpc. **a** PL assay for in situ detection of LRRK2 kinase activity (PL pS1292-LRRK2) (Cohort 2). Rotenone treatment elevated LRRK2 kinase activity (red), which was inhibited by treatment with 10-NO_2_-OA (45 mg/Kg) in DA neurons of SNpc (TH, blue—Scale bar: 35 μm). **b** Quantification of the PL pS1292-LRRK2 fluorescence signal. Symbols represent the normalized means of the intensity (with Vehicle set at 100%) from a single rat (6 slices/brain). Statistical analysis was performed by one-way ANOVA with post hoc Bonferroni correction (*p < 0.0001 compared to Vehicle, ^#^*p* < 0.0001 compared to Rotenone).

Kinase-activating mutations of LRRK2 are the most common cause of familial PD. Also, elevated LRRK2 kinase activity is found in the substantia nigra of iPD patients^16^, further implicating enhanced activity of LRRK2, independent of activating LRRK2 mutations, in PD pathogenesis. In the sub-acute rotenone model of PD in rat, there is a robust increase in LRRK2 activity (detected as pS1292-LRRK2 PL signal) in nigrostriatal dopaminergic neurons when compared to control rats. There was a dose-dependent reduction of LRRK2 activation in rotenone-treated rats given 5, 15, and 45 mg/Kg 10-NO_2_-OA (Supplementary Fig. 7), with the 45 mg/Kg dose of 10-NO_2_-OA maintaining levels of LRRK2 activity equivalent to control rats (Fig. 4 and Supplementary Fig. 7).

### 10-NO_2_-OA inhibits α-synuclein-mediated mitochondrial import impairment

**Fig. 5:**
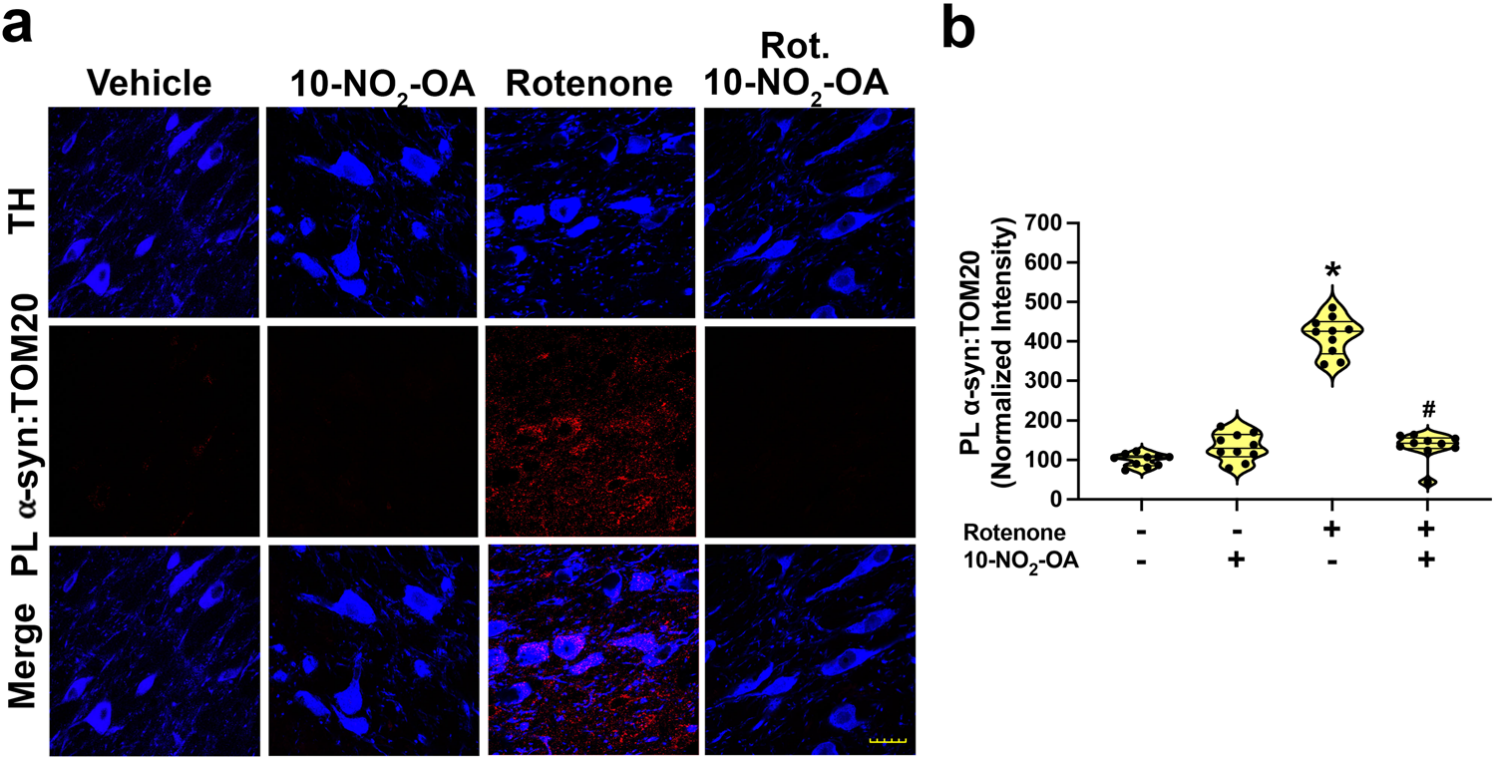
10-NO_2_-OA inhibits α-synuclein-mediated mitochondrial import impairment in a sub-acute rotenone model of PD. **a** Confocal microscopy analysis for PL signal between α-synuclein–TOM20 interaction in SNpc dopaminergic neurons (TH, blue) (Cohort 2). Rotenone treatment of rats induced a strong PL signal for α-synuclein:TOM20 (red). Co-treatment with 10-NO_2_-OA (45 mg/Kg) inhibited the rotenone-induced α-synuclein:TOM20 PL signal (Scale bar: 35 μm). **b** Quantification of the fluorescence signal. Symbols represent the normalized means of the intensity (with Vehicle set at 100%) from a single animal (6 slices /brain). Statistical analysis was performed by one-way ANOVA with post hoc Bonferroni correction (*p < 0.0001 compared to Vehicle, ^#^*p* < 0.0001 compared to Rotenone).

Post-translationally modified forms of α-synuclein bind to TOM20 to induce mitochondrial protein import impairment and mitochondrial dysfunction^18^. Sub-acute rotenone treatment induced α-synuclein-mediated mitochondrial protein import impairment as reflected by an increased α-synuclein:TOM20 signal in the SNpc of rats^18^. This rotenone-induced increase was dose-dependently inhibited by 10-NO_2_-OA (Supplementary Fig. 9). Of note, rotenone-induced α-synuclein:TOM20 interactions were also suppressed back to vehicle control levels by the administration of 45 mg/Kg 10-NO_2_-OA to rotenone-treated rats (Fig. 5 and Supplementary Fig. 9), reinforcing that 10-NO_2_-OA inhibits α-synuclein-mediated mitochondrial protein import impairment in dopaminergic neurons of SNpc.

### 10-NO_2_-OA limits microglial activation and neuroinflammation

**Fig. 6:**
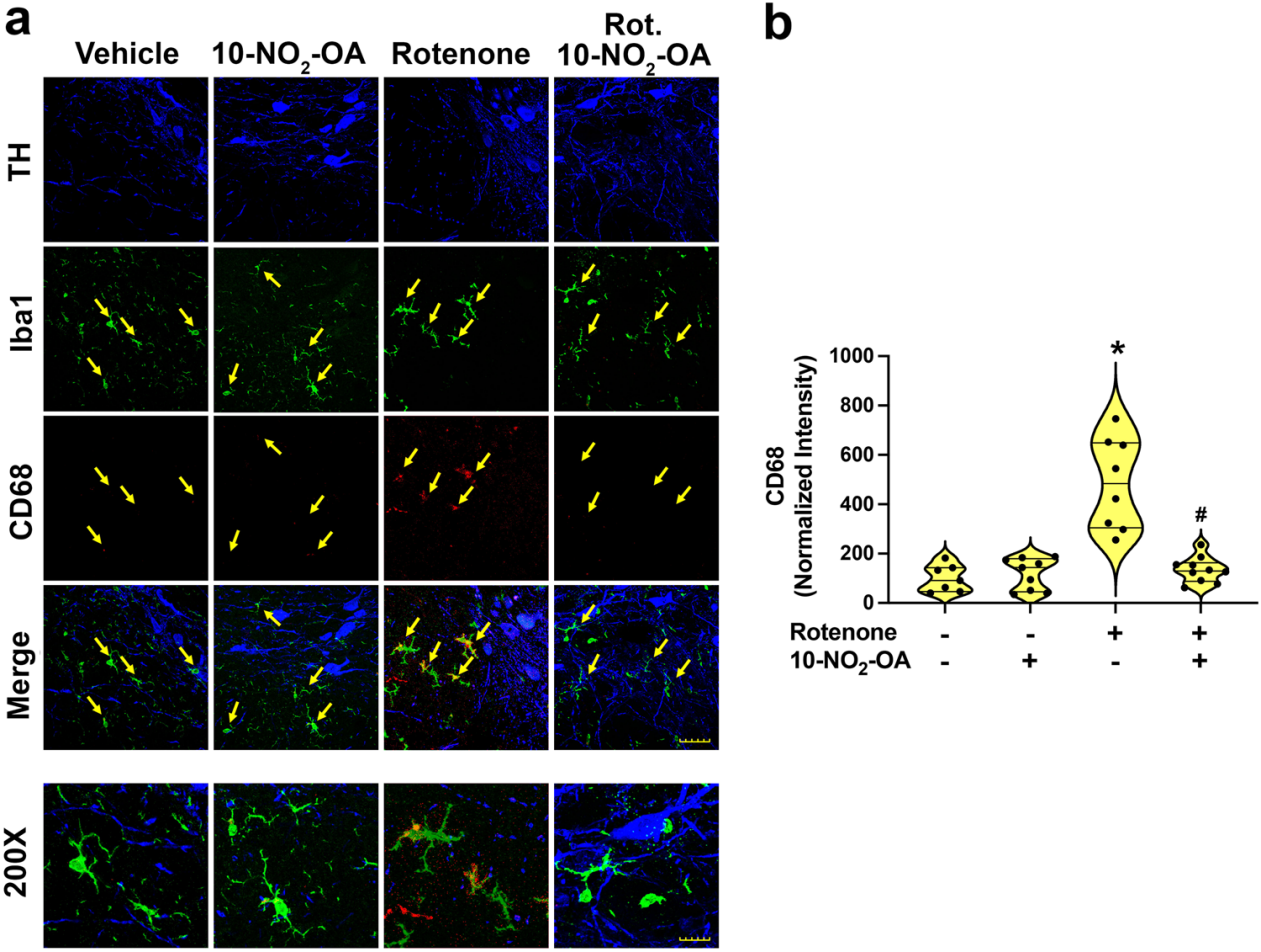
10-NO_2_-OA inhibits rotenone-induced microglial activation in SNpc (Cohort 2). **a** Rats treated with rotenone showed a significant increase in microglial activation (green) in SNpc analyzed immunohistochemically for the microglial activation marker, CD68 (red). Co-administration of 10-NO_2_-OA (45 mg/Kg) inhibited rotenone-induced microglial activation (Scale bar main figure: 35 μm; Scale bar ×200 magnification: 15 μm). **b** Quantification of the signal relative to CD68 in microglia (Iba1) with symbols that represent the normalized means of the intensity (with Vehicle set at 100%) from a single rat (4 slices/brain). The number of animals per treatment group were the following: Vehicle (*n* = 7), 10-NO_2_-OA (*n* = 9), Rotenone (*n* = 8), Rotenone + 10-NO_2_-OA (*n* = 10). Statistical analysis was performed by one-way ANOVA with post hoc Bonferroni correction (*p < 0.0001 compared to Vehicle and 10-NO_2_-OA, ^#^*p* < 0.0001 compared to Rotenone).

Upon activation, microglial cells induce neuroinflammatory responses that are linked with PD pathogenesis. To evaluate whether 10-NO_2_-OA modulates rotenone-induced PD-related neuroinflammatory responses, SNpc regions were immunohistochemically analyzed for (i) dopaminergic neurons (TH), (ii) microglia (Iba1), and (iii) the microglial activation marker CD68 (Fig. 6). Compared to vehicle and 45 mg/Kg 10-NO_2_-OA treatment groups, rotenone-treated rats showed a significant increased microglial activation as indicated by increased expression of CD68 in Iba1 (microglia)-positive cells. The administration of 10-NO_2_-OA (45 mg/Kg) significantly inhibited rotenone-induced microglial activation, with corresponding reduction of CD68-positive Iba1-positive cells, supporting potential anti-neuroinflammatory actions of 10-NO_2_-OA.

### 10-NO_2_-OA does not impact rotenone metabolism

To better define the neuroprotective actions of 10-NO_2_-OA in the rotenone-treated rat model of PD, potential effects of 10-NO_2_-OA on the metabolism of rotenone was measured in the brains of rotenone-treated rats by HPLC-MS/MS analysis. Both vehicle and 45 mg/Kg 10-NO_2_-OA treated rats had detectable rotenone in brains, while rats treated with 2.8 mg/Kg rotenone ± 45 mg/Kg 10-NO_2_-OA showed the same brain levels of rotenone, confirming that 10-NO_2_-OA did not induce any changes in rotenone pharmacokinetics (Supplementary Fig. 10).

## Discussion

A safe and effective drug strategy for preventing or slowing the progression of PD remains an unmet need. Recent clinical trials for “single-target” drugs and antioxidant strategies have consistently failed to decrease the progression of PD^7–10,46^. Multiple events, such as Nrf2-dependent gene expression, oxidative stress, α-synuclein aggregation, mitochondrial dysfunction, and neuroinflammation are understood to influence the pathogenesis of PD. This motivates the consideration of shifting from “one-drug, one-target” pharmacology to the evaluation of agents that can modulate the activity of multiple disease-relevant targets that might slow or even arrest the nigrostriatal neurodegeneration of PD. In this regard, electrophilic dimethyl fumarate (DMF) has shown therapeutic potential in PD and displays a multi-target mechanism of action beyond Nrf2 signaling, in the course of targeting the redox-sensitive-cysteine proteome^11,12,14,43^. This includes the modulation of NF-kB^47^, GAPDH^48^, and T-cell-mediated signaling^49^. DMF is primarily prescribed for the treatment of multiple sclerosis (MS), with side effects including flushing, diarrhea, nausea, vomiting, muscle cramping, and abdominal pain. A recent study showed that the majority of relapsing-remitting multiple sclerosis (RRMS) patients either discontinued DMF or switched to another therapy within 2 years^50^.

Electrophilic fatty acid nitroalkenes are endogenously generated by digestive and inflammatory reactions and are inactivated in a regulated manner by prostaglandin reductase-1^51^. The reversible reaction of electrophilic nitroalkenes with their primary biological target, the nucleophilic amino acid cysteine, engages a highly conserved population of hyper-reactive cysteine moieties that orchestrate an array of catalytic, redox signaling and protein structural functions^52^. This insight into redox-mediated cell regulation has accelerated studies into how the cysteinome transduces dietary and redox-derived chemical signals into biochemical and phenotypic responses and from this, new drug development strategies for treating complex pathologies.

The rationale behind the present evaluation of 10-NO_2_-OA as a PD therapeutic comes from the many broad and durable phenotypic responses that both natural and synthetic electrophiles can induce^53^. This has stimulated new investigation and development, with the synthesis and identification of 8561 different electrophilic protein modifiers, the documentation of 342 protein targets of electrophiles and >50 electrophilic FDA-approved covalent-modifying drugs^54^. Multi-target toxicity concerns, long held for covalent modifier agents, have been de-risked by an appreciation that organisms co-exist with and capitalize on diverse populations of endogenous electrophiles whose concentrations change in response to metabolic, inflammatory and environmental factors. These electrophilic species include dietary constituents (vegetable thiocyanates, oxo-fatty acids), metabolites of dietary polyphenols (e.g., resveratrol, curcumin), endogenous products of intermediary metabolism (fumarate, itaconate) and inflammatory mediators (multiple prostaglandins)^55–57^. Moreover, high throughput chemiproteomic methods are rapidly evolving to specifically identify the relatively limited population of endogenous proteins, as well as specific protein cysteine moieties, that are targeted by different small molecule electrophiles^58,59^.

The biosimilar fatty acid nitration product 10-NO_2_-OA is detectable endogenously^19,40^. Synthetic 10-NO_2_-OA has undergone 5 Phase I human safety studies and is being evaluated in an ongoing inflammation-related Phase II trial for the treatment of airway hyperreactivity in obese subjects (NCT03762395), with no reportable safety signals. The impact of oral nitroalkene administration in models of PD have not been evaluated. A potential neuroprotective role of 10-NO_2_-OA in PD was suggested by a report of nitro-fatty acid-induced PTM of α-synuclein and a decreased capacity of this modified α-synuclein to form insoluble fibrils^60^. Herein, we evaluated the potential neuroprotective actions of 10-NO_2_-OA in rotenone-treated N27-A dopaminergic neural cells and in a sub-acute rotenone rat model of PD which recapitulates many critical pathogenic events known to occur in human PD^6,16,17^.

The evaluation of 10-NO_2_-OA distribution and metabolism in the brain showed facile movement of electrophilic 10-NO_2_-OA across the BBB. A previous ^14^C-labeled 10-NO_2_-OA autoradiography-based pharmacokinetics study showed distribution of radioactive 10-NO_2_-OA in rat brain without differentiating between the active agent, its reduced inactive metabolite 10-NO_2_-SA, and possible β-oxidation products^61^. Herein, we analyzed brain tissue by HPLC-MS/MS to provide concentration of 10-NO_2_-OA and its reduced inactive product 10-NO_2_-SA.

One of the multiple transcriptional regulatory pathways impacted by 10-NO_2_-OA is the Keap1/Nrf2 regulation of antioxidant and cytoprotective responses, a potential target for treating chronic inflammatory and neurodegenerative diseases^43,62,63^. Analysis of post-mortem brains of PD and Alzheimer’s disease (AD) patients shows reduced Nrf2-dependent responses^64^. 10-NO_2_-OA activates Nrf2-dependent gene expression via the alkylation of Cys273 and Cys288 in the Nrf2 repressor Kelch-like ECH-associated protein-1 (Keap1). This PTM of Keap1 facilitates Nrf2 translocation to the nucleus and the activation of antioxidant response element (ARE)-mediated expression of diverse cytoprotective and repair proteins that induce anti-inflammatory and detoxification responses, thus limiting organ dysfunction in multiple animal models of inflammatory disease^27,35–38^. Based on these premises, it was affirmed that 10-NO_2_-OA activates cytoprotective Nrf2-dependent responses (activation of HO-1, Nqo-1, and Gclm expression) in N27-A dopaminergic cells. We observed no changes in Nrf2-related gene expression when N27-A cells were exposed to rotenone alone. Notably, rotenone mediated responses in vitro are influenced by the cell type, dosage of rotenone, and time of exposure^65–67^.

In agreement with our in vitro results, 10-NO_2_-OA-dependent Nrf2 activation was confirmed by the nuclear translocation of Nrf2 and the upregulation of the expression of the Nrf2 target gene HO-1 in the SNpc of rats, an area of the brain severely affected by PD. Instead, the downregulation of Nrf2 expression paralleled by a downregulation trend in HO-1 in rotenone-treated rats, was in agreement with some previous studies^13^ but not all^68^. This discrepancy can potentially be explained by the use of different rotenone dosages, exposure times, and by the experimental methodologies applied to these studies (qPCR and western blots in brain lysates) which may not provide cell-specific evidence. Our observations revealed differential cellular response in nigrostriatal DA neurons and provided further evidence of the widely recognized higher vulnerability of these neurons to oxidative stress. This motivated further exploration of the relative impact of Nrf2-induced neuroprotection in the context of nitroalkene modulation of other target responses in microglia, astrocytes, and non-dopaminergic neurons in other brain regions affected by PD.

The activity of NOX2, a superoxide and hydrogen peroxide-generating member of the NADPH oxidase family, is upregulated in the brain of PD patients and is linked with the pro-apoptotic and proinflammatory events occurring in PD^69,70^. Dopamine neurons of the SNpc display an exquisite vulnerability to oxidative stressors^4–6^. We recently reported that brain NOX2 is activated by mitochondrially derived ROS via a feedforward cycle that greatly amplifies ROS production. We demonstrated a central role of NOX2 in the amplification of pathogenic events linked with PD^15^, including the oxidative PTM of α-synuclein (4-HNE-α-synuclein, nitro-tyrosine-α-synuclein), LRRK2 kinase activation^16,71^ and autophagic defects implicated in the accumulation of toxic pSer129-α-synuclein^17,18^. This pathogenic cascade, modulated by multiple additional factors (e.g., kinase-enhancing LRRK2 mutations, α-synuclein expression levels, glucocerebrosidase (GBA) gene mutations, mitochondrial dysfunction, and NOX2-activating extracellular signaling) may be a scenario best addressed by a multi-target drug strategy that impacts diverse adaptive responses and multiple pathogenic mechanisms that contribute to pathogenesis of PD.

Oxidative stress promotes lipid peroxidation and formation of the irreversibly-reactive α,β-unsaturated hydroxyalkenal termed 4-HNE. In the brain, a key reaction target of 4-HNE is α-synuclein, leading to the formation of highly aggregable 4-HNE-α-synuclein adducts that are implicated in mitochondrial dysfunction, neuroinflammation, and nigrostriatal neurodegeneration^15^. 10-NO_2_-OA administration limited rotenone-induced 4-HNE accumulation and formation of 4-HNE-α-synuclein adducts in SNpc. Mitigation of oxidative stress and limitation of oxidative PTM of a-synuclein may thus contribute to the neuroprotective actions of 10-NO_2_-OA. In addition, biochemical studies of α-synuclein alkylation by 10-NO_2_-OA revealed a change in potential for fibrillization^60^. Further studies can help differentiate the relative impact of fatty acid nitroalkenes on modulating oxidative stress and the downstream pathogenic oxidative PTM of α-synuclein.

Kinase-activating mutations in the LRRK2 gene are strongly linked with familial PD^72^ and contribute to enhanced risk of idiopathic PD (iPD)^71^. It is also well established that LRRK2 kinase activation occurs in iPD independent of an activating mutation^16,73,74^. The present study reveals that 10-NO_2_-OA dose-dependently limits increases in rotenone-induced LRRK2 kinase activity in nigrostriatal dopamine neurons and may represent a central mechanism of action 10-NO_2_-OA in countering aspects of PD pathogenesis. Further studies will reveal whether this inhibition of LRRK2 kinase activity by 10-NO_2_-OA occurs by modification of vicinal redox-sensitive-cysteine residues (Cys2024 and Cys2025) in the kinase activation loop of LRRK2^75^ or by alternative mechanisms.

The cellular accumulation of pathogenic α-synuclein PTM products can impact mitochondrial protein import function by interacting with the receptor TOM20^18^.

This includes oxidized lipid-α-synuclein adducts that have oligomerized and pS129-α-synuclein, resulting from LRRK2-mediated endo-lysosomal impairment. The inhibition of the rotenone-induced α-synuclein:TOM20 interaction on mitochondria by 10-NO_2_-OA may be due to reduced formation of the various α-synuclein PTM products that interact with TOM20. Upstream, this may be a consequence of the ability of 10-NO_2_-OA to suppress activation of NOX2 and LRRK2.

There is broad consensus that neuroinflammation is important in PD^76^ and that Nrf2-regulated gene expression plays a crucial role in the modulation of microglial dynamics. Impairment of Nrf2 signaling elicits microglial activation and exacerbation of neuroinflammation, as reflected by the upregulation of multiple indices of inflammation and downregulation of anti-inflammatory mediators^77^. The inhibition of microglial activation induced by 10-NO_2_-OA in SNpc may be explained by the concerted activation of Nrf2-regulated adaptive gene expression and the inhibition of nuclear factor-kappa B (NF-kB)-regulated proinflammatory signaling^29,39,78–80^. The transcription factor NF-kB has a central role in neuroinflammation, and its activation with nuclear translocation of the RelA (p65) subunit in neurons and glia has been reported in human PD postmortem brains and in animal models^81,82^. Thus, the suppression of NF-kB signaling by small molecule nitroalkenes is potentially beneficial in PD therapy^82^. Such inhibition can be a consequence of (a) 10-NO_2_-OA alkylation of Cys38 and Cys105 in the p65 subunit which, in turn, inhibits DNA binding, (b) promotion of p65 polyubiquitination and proteasomal degradation, and/or (c) inhibition of the inhibitor of NF-kB subunit kinase β^29,39,78,80^. Future studies are planned to define the specific mechanisms of neuronal cell NF-kB inhibition in the context of PD therapy.

In summary, we report that the reversibly-reactive fatty acid nitroalkene 10-NO_2_-OA is effective both in vitro and in vivo model systems showing the limitation of multiple pathogenic events that have been proposed to contribute to the pathogenesis of PD. Future studies are aimed at defining more specific molecular mechanisms of neuroprotection lent by this multi-target agent. In addition, we will determine whether fatty acid nitroalkenes can prevent nigrostriatal neurodegeneration in more chronic PD models that also manifest the impaired motor functions characteristic of PD. Given the human safety of 10-NO_2_-OA, these findings might lead to a safely deployable therapeutic approach for alleviating the clinical and economic burden of PD.

## Methods

### Study design

This study was designed to define the neuroprotective actions of 10-NO_2_-OA in the in vivo rotenone model of PD and the mechanisms of action of this nitroalkene in rotenone treated N27-A dopaminergic cells. All in vitro experiments were replicated at least three times. In vivo experiments using rotenone treatment, with or without concomitant 10-NO_2_-OA treatment, were performed in rats, and outcomes were analyzed by blinded assessors. Rats were randomized to treatment group. There was no exclusion of outliers.

### Reagents

All reagents were purchased from Sigma–Aldrich, unless otherwise specified. Synthesis of (*E*)-10-[^15^N]nitro-[15,15,16,16-D_4_]octadec-9-enoic acid (10-NO_2_-[^15^N/D_4_]OA) and (*E*)-10-[^15^N]nitro-[15,15,16,16-D_4_]octadecanoic acid (10-NO_2_-[^15^N/D_4_]SA) was performed as previously^83,84^. 2-isopropyl-8,9-dimethoxy-1,2,12,12a-4h-6ah-chromeno(3,4-b)furo(2,3-h)chromen-6-one, and poly-D-lysine hydrobromide were purchased from Sigma-Aldrich (St. Louis, MO). Solvents used for extractions and mass spectrometric analyses were from Burdick and Jackson (Muskegon, MI). Pen-Strep was purchased from Gibco^TM^-Thermo Fisher Scientific (Waltham, MA), RPMI 1640 media with L-glutamine from Corning (Corning, NY), 3-(4,5-dimethylthiazol-2-yl)-2,5-diphenyltetrazolium bromide (MTT) solution and TRIzol reagent from Invitrogen (Carlsbad, CA), Mygliol 812 (oil rich in saturated medium chain triglycerides, IOI Oleochemical Pharma). For a complete list of antibodies used in immunostaining analysis see Supplementary Table 1.

### Cell cultures

N27-A rat dopaminergic neural cells (provided by Dr. Curt Freed-University of Colorado)^42^ were cultured in poly-D-lysine hydrobromide coated well plates with RPMI 1640 medium with L-glutamine 10% fetal bovine serum (FBS), 100 U/mL Pen-Strep and maintained at 37 °C, and 5% CO_2_. When cells reached confluency, the medium was replaced with RPMI 2% FBS to minimize FBS-related scavenging of 10-NO_2_-OA and treatments were performed over 24 h. Then, cells were harvested or fixed to perform the assays.

### Cell viability

Viability of N27-A cells was evaluated by MTT assay. Briefly, cells were seeded on poly-D-lysine coated 96-well plates at a density of 14,000 cells/100 µL well in RPMI 2% FBS. Then, 10 μL 5 mg/mL MTT solution was added, and cells were incubated at 37 °C for 2 h. After incubation, the media was replaced with 100 μL ethanol/DMSO (1:1, v/v) and cells were left in the dark for 10 min at room temperature. Absorbance at 540 nm, characteristic of a purple formazan product generate by viable cells, was measured using a plate reader (SpectraMax M2).

### Quantitative real-time PCR

RNA was extracted from N27-A cells with TRIzol reagent according to the manufacturer’s protocol. RNA concentration was measured with NanoDrop 2000 (Thermo Fisher) and 1 μg of RNA template was utilize for cDNA transcription using an iScript cDNA Synthesis Kit (Bio-Rad, 1708891 BUN). RT-qPCR was performed on StepOnePlus Real-Time PCR system using TaqMan Fast Advanced Master mix to quantify expression of gene of interest: *Hmox1* (Rn00561387_m1), *Nqo1* (Rn00566528_m1), *Gclm* (Rn00568900_m1), all from Applied Biosystems. Data are normalized to rat endogenous control *Actin* (Rn00667869_m1), from Applied Biosystems.

### Animal study and brain collection

The neuroprotective signaling actions of 10-NO_2_-OA were assessed using an established sub-acute rotenone model of PD in rat^15,16^. First, we performed a 10-NO_2_-OA dose-dependent pilot study (cohort 1) treating six groups of male aged (8–9 months, ~ 450 g) Lewis rats (Envigo) for 5 days with: (i) Vehicle (Mygliol 812) (*p.o*.) (*n* = 2), (ii) 10-NO_2_-OA 45 mg/Kg (*p.o*.) (*n* = 2), (iii) Rotenone 2.8 mg/Kg (*i.p*.) (*n* = 3), (iv, v, and vi) Rotenone 2.8 mg/Kg (*i.p*.) + 10-NO_2_-OA at 5, 15, and 45 mg/Kg (*p.o*.) (*n* = 3 per group). This pilot study provided preliminary evidence that 10-NO_2_-OA dose-dependently induced neuroprotection and allowed to determine the most effective oral dose for the final study (cohort 2) with a higher number of animals per group (*n* = 10) as follow: (1) Vehicle (Mygliol 812) (*p.o*.), (2) 10-NO_2_-OA 45 mg/Kg (*p.o*.), (3) Rotenone 2.8 mg/Kg (*i.p*.), (4) Rotenone 2.8 mg/Kg (*i.p*.) + 10-NO_2_-OA 45 mg/Kg (*p.o*.). The number of animals was determined from repeated power analyses of sub-acute (5 days) rotenone model. Using data from previous studies, power analyses typically suggest 7–10 animals per group to be able to detect a ~20% difference compared to control for rotenone model. To evaluate the rapidity of Nrf2 pathway engagement in brains, rats were treated and then sacrificed for analysis 5 h later. Rats were deeply anesthetized with sodium pentobarbital (*i.p*), bled to death, and residual blood was washed out after 15 min by trans-cardiac perfusion of 150 mL cold phosphate buffered saline. Then, rats were decapitated with a guillotine and brains were immediately removed and cut in half along the mid-sagittal suture. Half brain was fixated with 4% paraformaldehyde (PFA) for immunostaining studies while the other half was flash-frozen and stored at −80 °C until used for HPLC-tandem mass spectrometry (HPLC-MS/MS) analysis.

In vivo experiments were performed accordingly with the protocols approved by the University of Pittsburgh Institutional Animal Care and Use Committee (IACUC). The Division of Laboratory Animal Resources (DLAR) at the University of Pittsburgh provided appropriate veterinary care to the animals monitoring them twice daily for behavior and overall health throughout the experiments. Euthanasia was performed accordingly with the recommendations of the Panel on Euthanasia of the American Veterinary Medical Association.

### Immunohistochemistry

Immunohistochemistry was performed according to standard procedures^85^. 4% paraformaldehyde post-fixed brain specimens were placed in 30% sucrose. Thirty-five micrometers thick free-floating sections were collected using a microtome and stored in antifreeze at −20 °C until use. For each experiment, all tissues were stained at the same time. After a blocking step in 10% serum, sections were incubated 48 h with primary antibodies. Fluorescently labeled secondary antibodies were used for detection (Supplementary Table 1).

### Proximity ligation assays

Proximity ligation assay (PLA) (DUO92004-100RXN; DUO92002-100RXN; DUO92007-100RXN, Sigma-Aldrich) was performed in 4% PFA–fixed tissue or cells as previously described^15,16,18,85^. Samples were incubated with specific primary antibodies to the proteins to be detected. Secondary antibodies conjugated with oligonucleotides (provided in the Sigma kit) were added to the reaction and incubated. Ligation solution, consisting of two oligonucleotides and ligase, was added. In this assay, the oligonucleotides hybridize to the two proximity ligation probes and join to a closed loop if they are in proximity. Amplification solution, consisting of nucleotides and fluorescently labeled oligonucleotides, was added together with polymerase. The oligonucleotide arm of one of the proximity ligation probes acts as a primer for “rolling-circle amplification” using the ligated circle as a template, and this generates a concatemeric product. Fluorescently labeled oligonucleotides hybridize to the rolling-circle amplification product. The proximity ligation signal was visible as a distinct fluorescent spot and was analyzed by confocal microscopy. Experimental controls for NOX2 and LRRK2 PLAs were run in parallel by primary antibodies deletion^85^ (Supplementary Fig. 8). All information (catalog numbers and dilutions) regarding the antibodies used for the PLAs can be found in Supplementary Table 1.

### Confocal microscopy and imaging parameters

Fluorescent images were taken using an Olympus BX61 confocal microscope and Fluoview 1000 software (Melville, NY). A negative control (secondary antibody only) slide was prepared for each experiment to subtract the background fluorescence. Imaging parameters were monitored to ensure that images were above background level and below pixel saturation. For quantitative comparisons between groups, all imaging parameters (e.g., laser power, exposure, and pinhole) were held constant across specimens. Confocal images were captured using either a UPlansApo ×60/1.35 oil or ×100/1.40 oil objective lens. A minimum of three images per tissue section and 3 tissue sections per animal were used for each analysis. Each image contained ~6–20 neurons per image; therefore, 54–180 neurons per animal were analyzed. Depending on the variability of each stain, 4–10 animals were analyzed for each confocal experiment.

### Analysis of free NO_2_-FA in brain

Frozen brain samples (cohort 2) were finely pulverized under dry ice conditions using the CellCrusher^®^ (Schull, Ireland) and stored at −80 °C. Brain powder (~250 mg) was spiked with 0.5 pmol internal standards (I.S.) mix 10-NO_2_-[^15^N/D_4_]OA and 10-NO_2_-[^15^N/D_4_]SA, and free NO_2_-FA were extracted with 500 µL acetonitrile. Then, samples were vortexed for 60 s, centrifuged at 21,300 × *g* for 10 min at 4 °C, and the supernatants were dried under a flow of nitrogen, resuspended into 70 µL acetonitrile and analyzed by HPLC-MS/MS.

### Analysis of rotenone

Brain powder (cohort 2) (~10 mg) was spiked with 6.3 pmol internal standard (I.S.) 2-isopropyl-8,9-dimethoxy-1,2,12,12a-4h-6ah-chromeno(3,4-b)furo(2,3-h) chromen-6-one and rotenone was extracted with 300 µL acetonitrile. Then, samples were sonicated for 5 min, vortexed for 30 s, centrifuged at 20,000 × *g* for 5 min at 4 °C, and the supernatants were analyzed by HPLC-MS/MS.

### HPLC-MS/MS analysis

Analysis of NO_2_-FA and rotenone was performed with a C18 Luna column (2 × 20 mm, 5 µm, Phenomenex), and chromatographically resolved with a flow rate of 0.7 mL/min and a gradient solvent system consisting of water 0.1% acetic acid (solvent A) and acetonitrile 0.1% acetic acid (solvent B). The gradient program was: 30–100% solvent B (0–3 min); 100% solvent B (0.6 min) followed by 1.4 min re-equilibration to initial conditions. 10-NO_2_-OA and its metabolite 10-NO_2_-SA were detected using a QTRAP 6500+ triple quadrupole mass spectrometer (Sciex, Framingham, MA) in negative mode with the following parameters: declustering potential (DP) − 50 V, collision energy (CE) − 42 eV, entrance potential (EP) and collision cell exit potential (CXP) − 5 V, and a temperature of 650 °C. Concentrations in rat brain were obtained using calibration curves in presence of labeled internal standards following the selected MRM transitions: MRM 326.2/46 for 10-NO_2_-OA and MRM 331.2/47 for its I.S. 10-NO_2_-[^15^N/D_4_]OA, while MRM 328.2/46 for 10-NO_2_-SA and MRM 333.2/47 for its corresponding I.S. 10-NO_2_-[^15^N/D_4_]SA. Results are reported as fmol/mg brain.

Rotenone was detected using an API 5000 triple quadrupole mass spectrometer (Applied Biosystems, San Jose, CA) equipped with an electrospray ion source (ESI) in positive mode with the following parameters: DP 90 V, EP 10 V, CXP 14 V, and a temperature of 700 °C. The following transitions and CE were used: 395.3/367.2, 395.3/213.2, 395.3/192.2 for rotenone, while 397.3/369.2, 397.3/215.2, 397.3/192.2 for I.S. with CE 30, 32, and 35 eV respectively. Levels of rotenone in rat brain were obtained using selected MRM transitions for rotenone (MRM 395.3/192.2) and its I.S. (MRM 397.3/192.2). Results are reported as area ratio rotenone/I.S. normalized per mg brain powder.

### Statistical analysis

Values are expressed as means ± standard deviation and statistical analysis was performed by one-way ANOVA with post hoc Bonferroni correction (*p < 0.05). Statistical comparisons between two groups have been performed by paired *t*-test.

### Reporting summary

Further information on research design is available in the Nature Portfolio Reporting Summary linked to this article.

## Supplementary information

Supplementary material

Reporting summary

## Data Availability

The datasets generated during and/or analyzed during the current study are available from the corresponding author upon request.
