# Supplementary material for: Neuroprotective actions of a fatty acid nitroalkene in Parkinson’s disease
Source: NPJ Parkinsons Dis. 2023 Apr 7;9:55. doi: 10.1038/s41531-023-00502-3 (PMC10082007; doi:10.1038/s41531-023-00502-3)
Supplement: Supplementary file 1 — Supplementary material [file 41531_2023_502_MOESM1_ESM.pdf]

Supplementary Material

Neuroprotective actions of a fatty acid nitroalkene in Parkinson's disease.

Roberto Di Maio[a,b*], Matthew T. Keeney[a,b,c], Veronika Cechova[c], Amanda Mortimer[a,b], Ahssan Sekandari[c], Pascal Rowart[c], J. Timothy Greenamyre[a,b], Bruce A. Freeman[c], Marco Fazzari[c*]

[a] Pittsburgh Institute for Neurodegenerative Diseases, [b] Department of Neurology, University of Pittsburgh, Pittsburgh, PA, 15213, U.S.A. [c] Department of Pharmacology and Chemical Biology, University of Pittsburgh, 200 Lothrop Street, Pittsburgh, PA, 15261, U.S.A.

*****Corresponding Authors:
R. Di Maio: rod16@pitt.edu
M. Fazzari: maf167@pitt.edu

Supplementary Table 1. Antibodies used in this study.

Supplementary Figure 1. Cytotoxicity of 10-NO_2-OA in N27-A dopaminergic cells.

Supplementary Figure 2. 10-NO_2-OA prevents rotenone-induced NOX2 and LRRK2 activation and oxidative stress in vitro.

Supplementary Figure 3. 10-NO_2-OA activates Nrf2-dependent gene expression in rotenone treated N27-A dopaminergic cells.

Supplementary Figure 4. Analysis of free 10-NO_2-OA and its inactive metabolite in rat brain (cohort 2).

Supplementary Figure 5. 10-NO_2-OA inhibited lipid peroxidation (4-HNE) in the subacute PD model of rotenone.

Supplementary Figure 6. 10-NO_2-OA inhibited NOX2 activation in SNpc of rat rotenone model.

Supplementary Figure 7. 10-NO_2-OA prevented rotenone-induced LRRK2 activation in SNpc.

Supplementary Figure 8. Validation of PL assays for LRRK2 and NOX2 by antibody depletion.

Supplementary Figure 9.10-NO_2-OA inhibits α-synuclein-mediated mitochondrial import impairment in the subacute PD model of rotenone.

Supplementary Figure 10. Analysis of rotenone in whole rat brain (cohort 2).

Host	Antibody	Catalog #	Vendor	Dilution	Use
Mouse	NOX2/gp91^{phox}	ab80897	Abcam	1:1000 (ICC)<br>1:500 (IHC)	PLA NOX2 activity
Rabbit	NCF1[FPR13131-25] (p47^{phox})	ab181090	Abcam	1:1000 (ICC)<br>1:500 (IHC)	PLA NOX2 activity
Mouse	α-synuclein	610787	BD Transduction	1:1000 (ICC)<br>1:500 (IHC)	PLA 4-HNE–α-syn PLA α-syn–TOM20
Rabbit	4-hydroxynonenal	ab46545	Abcam	1:1000 (ICC)<br>1:500 (IHC)	PLA 4-HNE–α-syn 4-HNE IHC
Rabbit	TOM20	ab186735	Abcam	1:500 (IHC)	PLA α-syn–TOM20
Rabbit	LRRK2 (pS1292)	ab203181	Abcam	1:1000 (ICC)<br>1:500 (IHC)	PLA LRRK2 activity
Mouse	LRRK2 (N241A)	75-253	UC Davis	1:1000 (ICC)<br>1:500 (IHC)	PLA LRRK2 activity
Sheep	Tyrosine Hydroxylase	AB1542	Millipore	1:2000 (IHC)	DA neurons marker
Rabbit	Iba1	019-19741	Wako	1:500 (IHC)	Microglial marker
Mouse	Heme Oxigenase-1 (HO-1)	MA1-112	Thermo Fisher Scientific	1:250 (IHC)	HO-1 IHC
Rabbit	Nrf2	Sc-722	Santa Cruz	1:500 (IHC)	Nrf2 IHC

Supplementary Table 1. Antibodies used in this study.

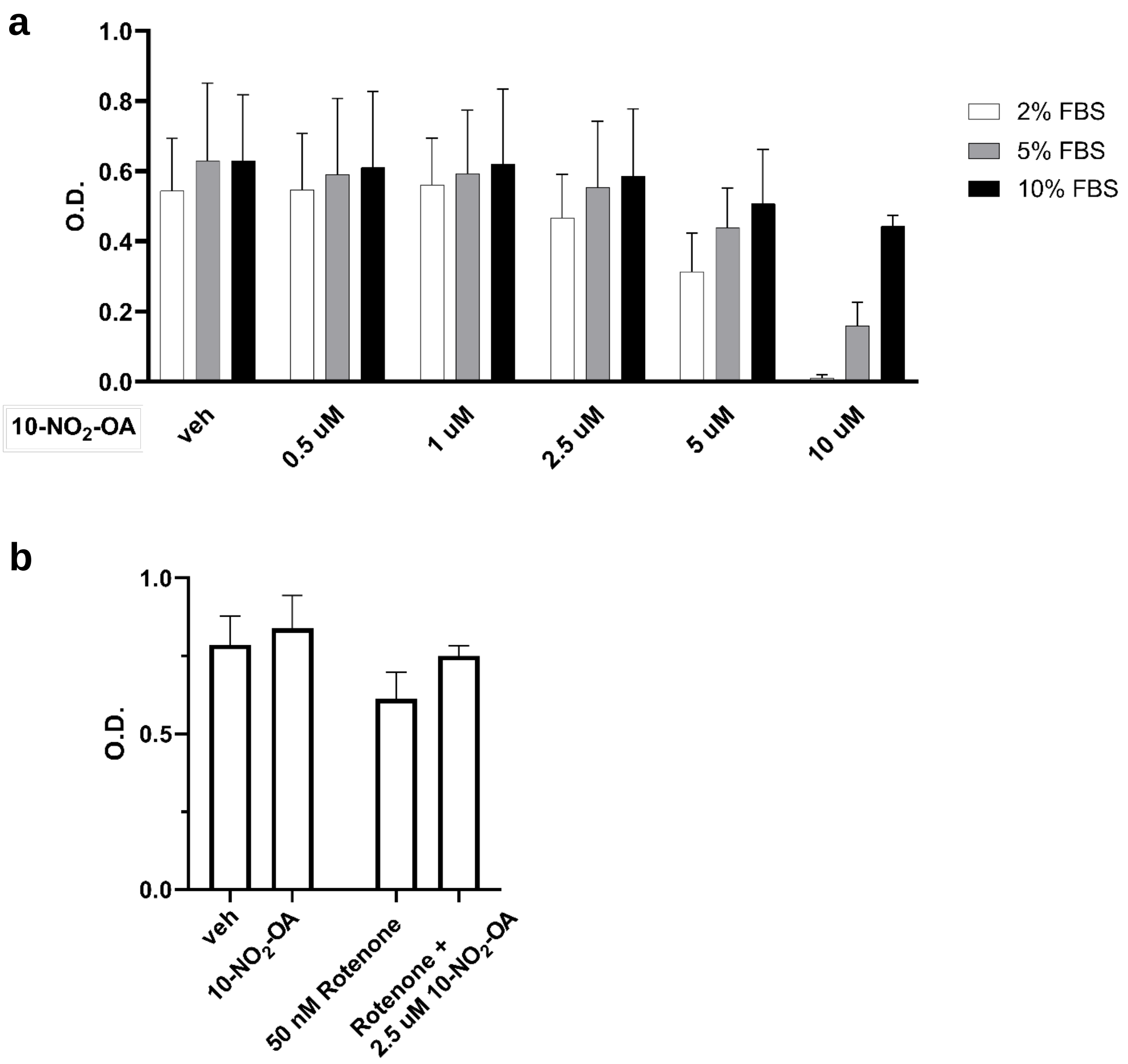

Supplementary Figure 1. Cytotoxicity of 10-NO_2-OA in N27-A dopaminergic cells. Cell viability was assessed by MTT assay and recorded by optical density (O.D.). N27A dopaminergic cells were exposed for 24 hours to: (a) 2-5-10% FBS, Vehicle and increasing concentrations of 10-NO_2-OA (0.5 µM-10 µM). (b) Vehicle, Rotenone (50 nM) and Rotenone (50 nM) + 10-NO_2-OA (2.5 µM) in 2% FBS. All data represents the mean ± SD of at least three independent experiments with n=3.

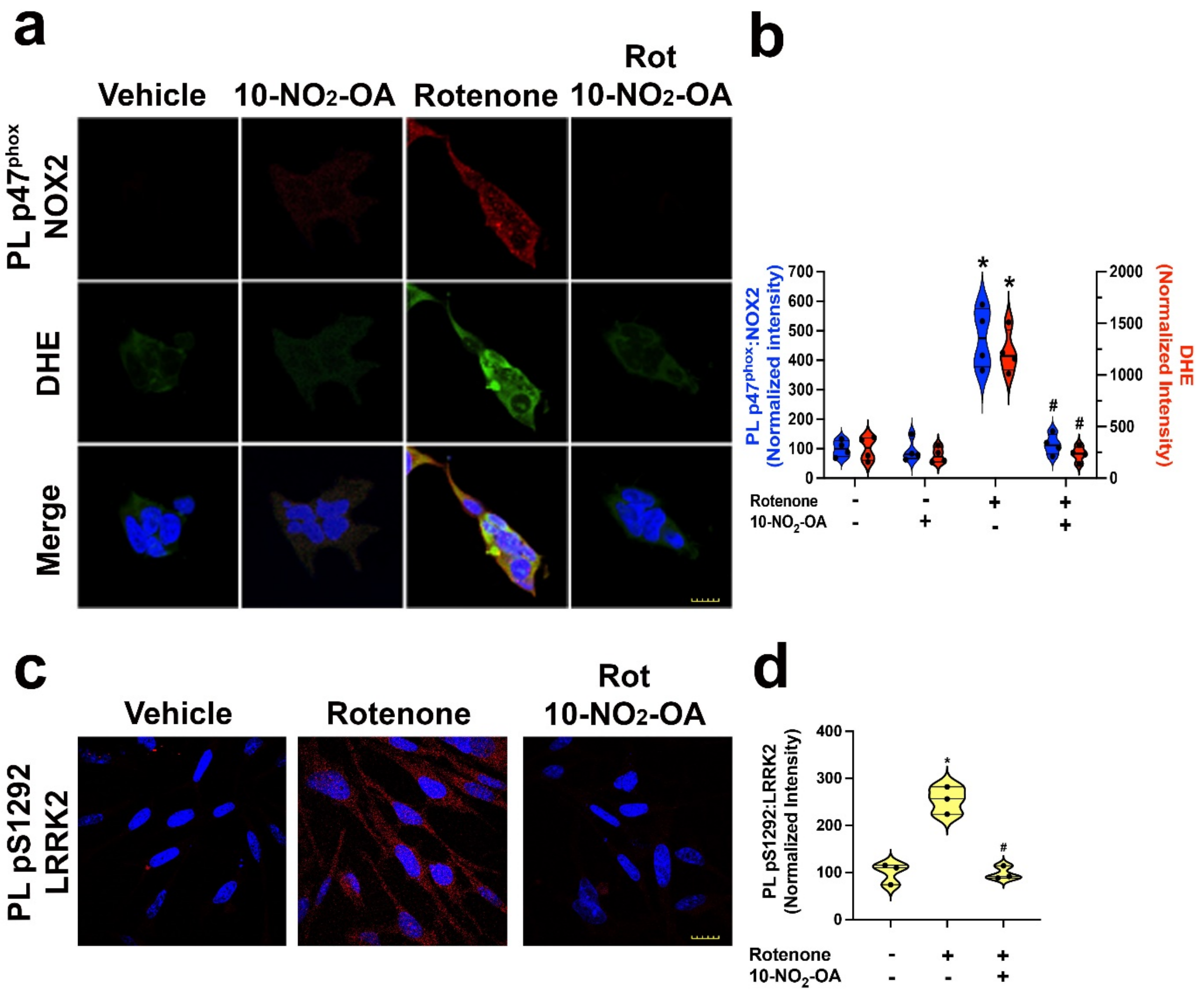

Supplementary Figure 2. 10-NO_2-OA prevents rotenone-induced NOX2 and LRRK2 activation and oxidative stress *in vitro*. N27A dopaminergic cells were exposed for 24 hours to Vehicle, Rotenone (50 nM) and Rotenone (50 nM) + 10-NO_2-OA (2.5 µM). (a) Rotenone elicited an increase in NOX2 activation as detected PL $p47^{phox}$:NOX2 (red). This was accompanied by an increase in cytoplasmic superoxide measured by DHE (green). Both NOX2 activation and DHE were prevented by co-treatment with 10-NO_2-OA (Scale bar: 5µm). (b) Quantitative analysis of PLA-related fluorescence intensity. (c) Co-treatment with 10-NO_2-OA prevented rotenone mediated LRRK2 activation (PL pS1292:LRRK2, red - Scale bar: 30µm). (d) Quantitative analysis of PLA-related fluorescence intensity. Each symbol represents average of normalized signal (with Vehicle set at 100%) from a single independent experiment. Statistical testing by ANOVA with post hoc Bonferroni correction (*, p<0.0001 compared to Vehicle, #, p<0.0001 compared to Rotenone).

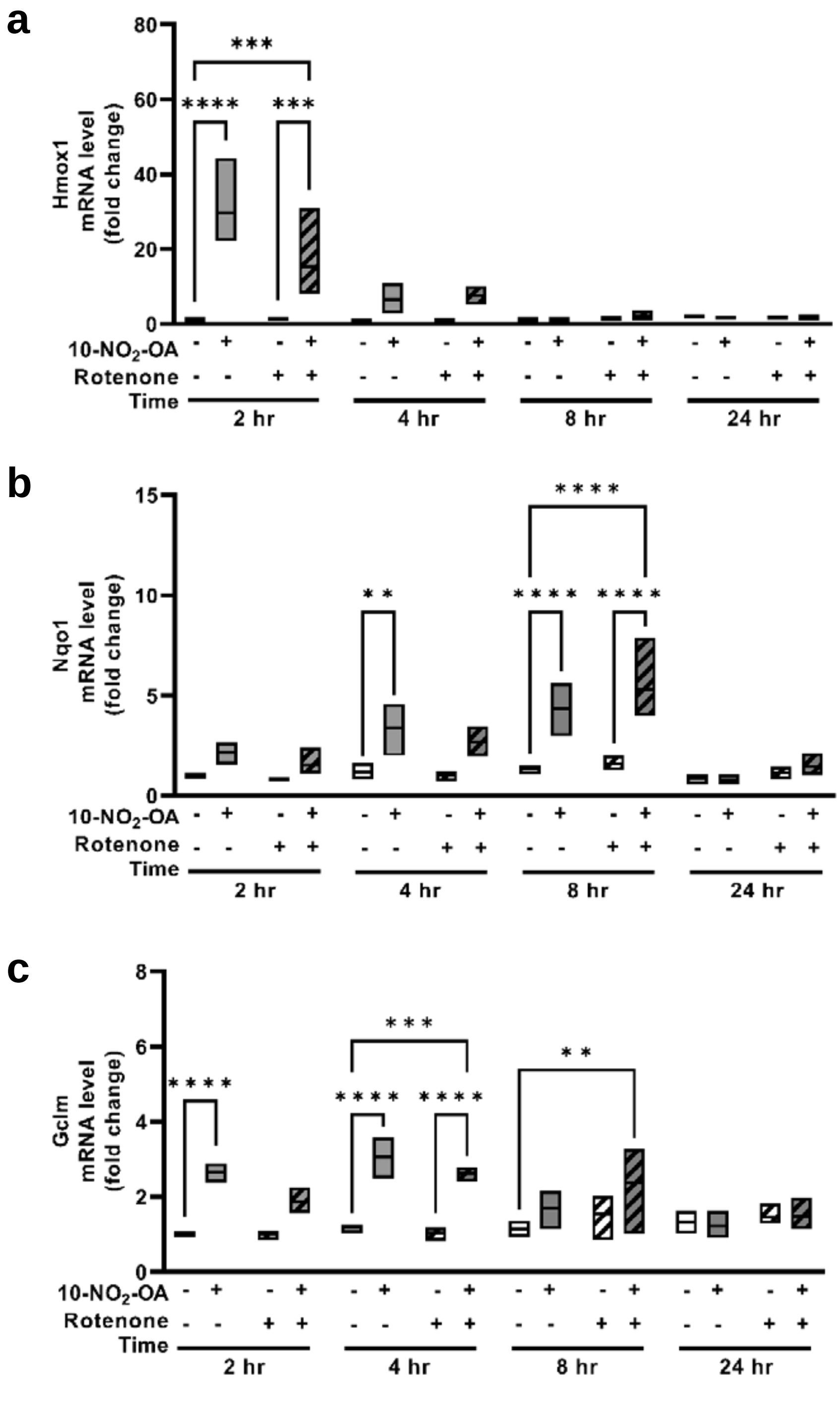

Supplementary Figure 3. 10-NO_2-OA activates Nrf2-dependent gene expression in rotenone treated N27-A dopaminergic cells. Relative mRNA levels of the following Nrf2 target genes: (a) heme oxygenase-1 (Hmox1, HO-1), (b) NAD(P)H dehydrogenase quinone-1 (Nqo-1), and (c) glutamate–cysteine ligase modifier subunit (Gclm), after treatment with 10-NO_2-OA (2.5 μM) and Rotenone (50 nM) for 2, 4, 8 and 24 hours in N27-A rat dopaminergic neural cell line. Values represent the range (minimum-maximum)

and mean of 4 independent experiments n=1 with statistical significance performed by two-way ANOVA with post hoc Bonferroni correction indicated by **** p ≤ 0.0001, *** p ≤ 0.001, and ** p ≤ 0.01.

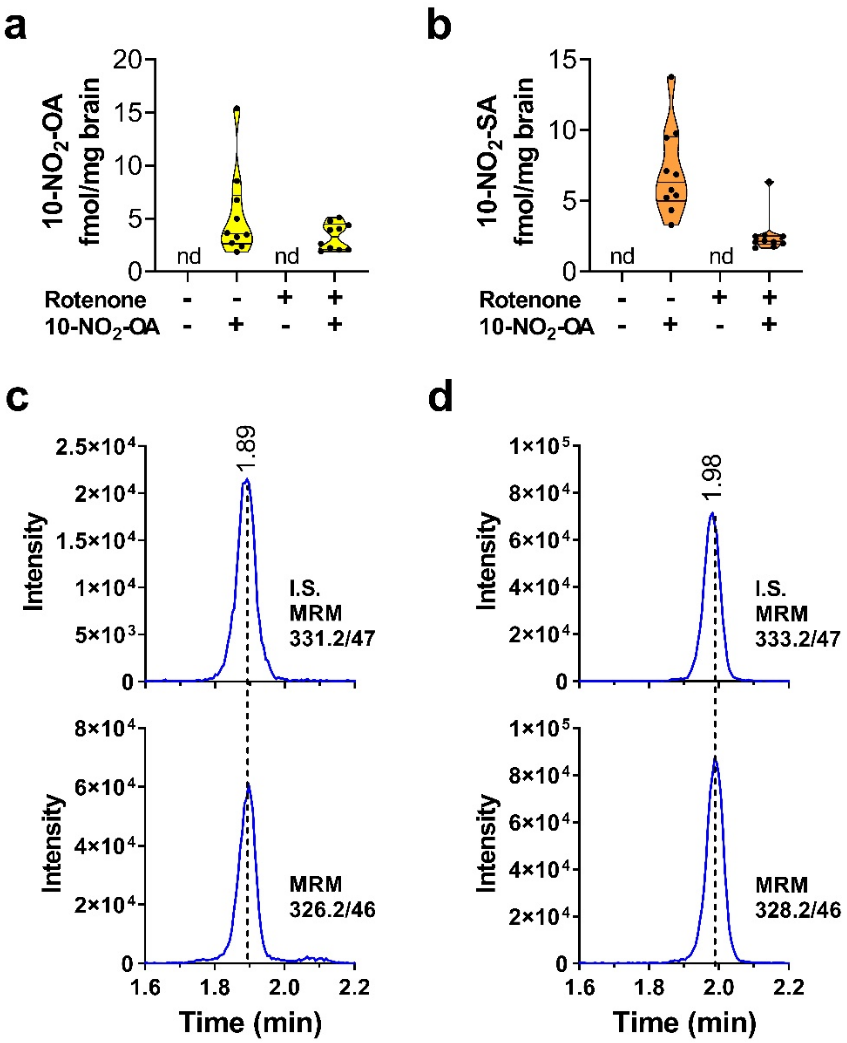

Supplementary Figure 4. **Analysis of free 10-NO_2-OA and its inactive metabolite in rat brain (cohort 2).** Brain concentrations of (a) 10-NO_2-OA, and (b) 10-NO_2-SA, five hours after the last treatments, were obtained by HPLC-MS/MS analysis using calibration curves in presence of the labeled internal standards 10-NO_2-[$^{15}N/D_4$]OA and 10-NO_2-[$^{15}N/D_4$]SA respectively. Representative extracted ion chromatograms in rat brain treated with Rotenone + 10-NO_2-OA, (c) the internal standard 10-NO_2-[$^{15}N/D_4$]OA (upper panel, MRM 331.2/47) and 10-NO_2-OA (lower panel, MRM 326.2/46), and (d) the internal standard 10-NO_2-[$^{15}N/D_4$]SA (upper panel, MRM 333.2/47) and 10-NO_2-SA (lower panel, MRM 328.2/46). nd = not detected.

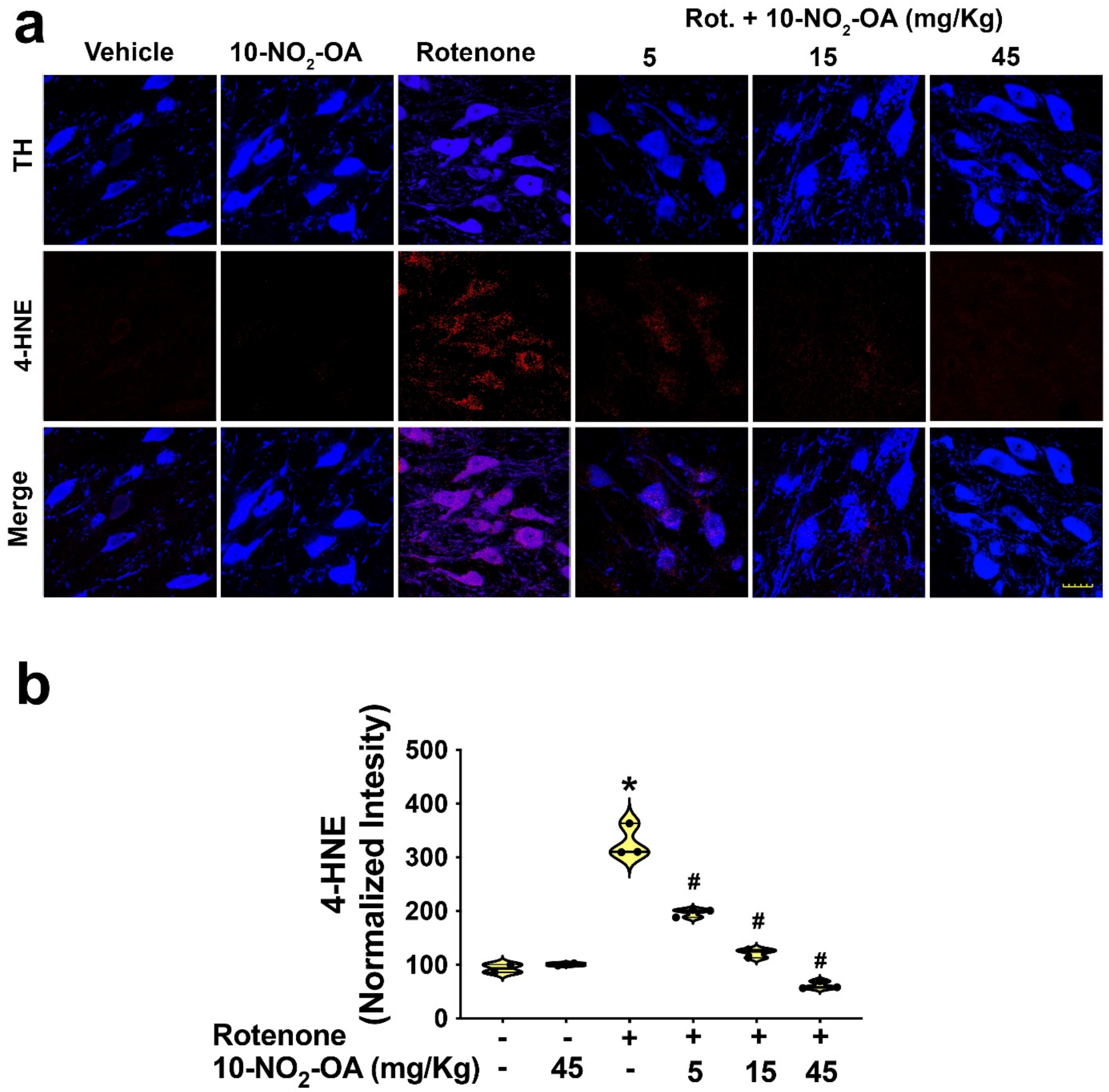

Supplementary Figure 5. 10-NO_2-OA inhibited lipid peroxidation (4-HNE) in the subacute PD model of rotenone. (a) 4-HNE immunostaining (red) in dopaminergic neurons (TH, blue) in the SNpc of rats (cohort 1 – Scale bar: 30μm) with below correspondent quantifications of the fluorescence signal (b). Symbols represent the normalized means of the intensity (with Vehicle set at 100%) from a single animal (6 slices /animal). Statistical analysis was performed by one-way ANOVA with post hoc Bonferroni correction (*, $p<0.0001$ compared to Vehicle, #, $p<0.0001$ compared to Rotenone).

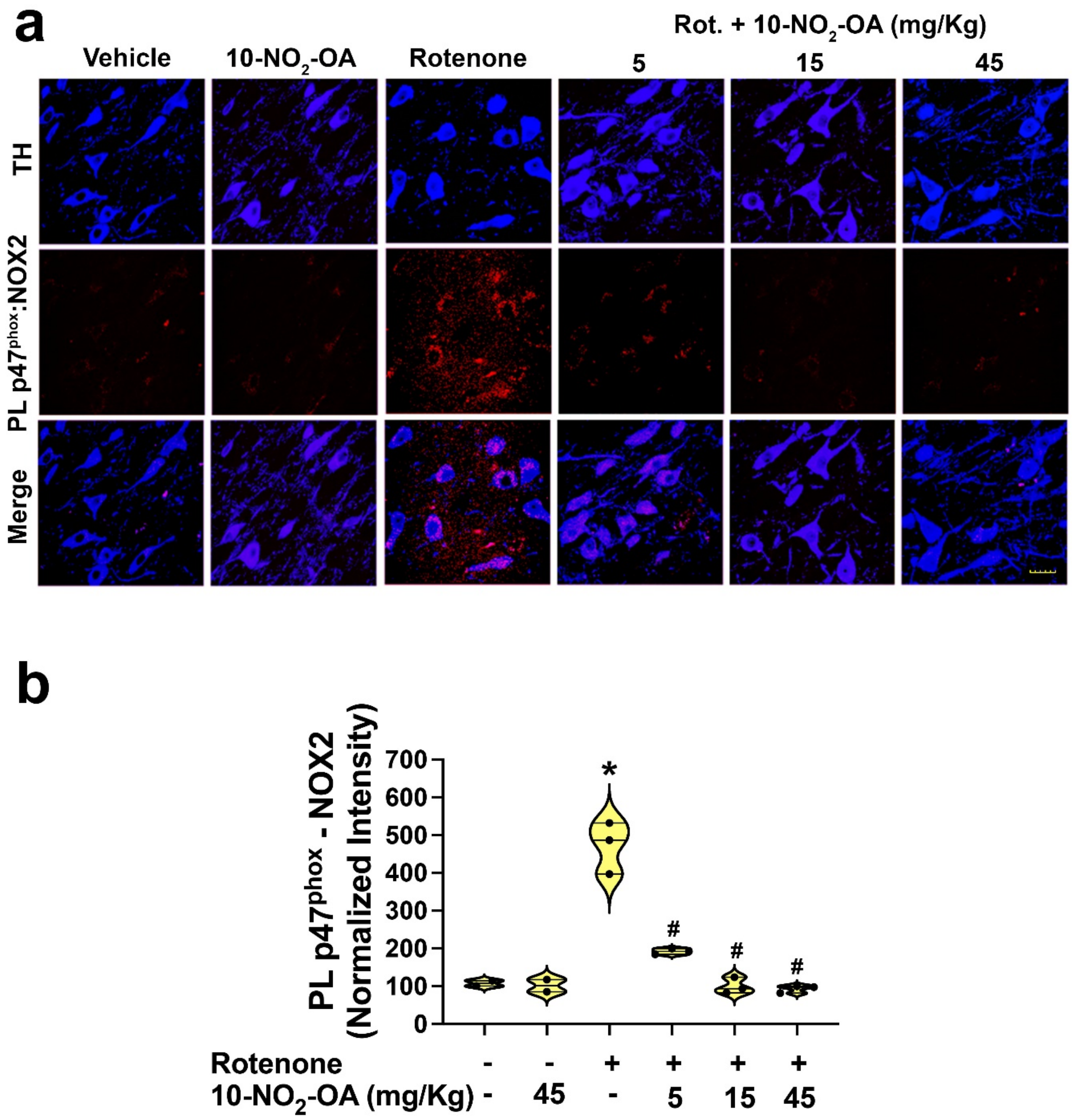

Supplementary Figure 6. 10-NO₂-OA inhibited NOX2 activation in SNpc of rat rotenone model. (a) PL p47^{phox}:NOX2 (red) in dopaminergic neurons (TH, blue) in the SNpc of cohort 1 rats (Scale bar: 30μm). (b) Quantifications of the fluorescence signal. Symbols represent the normalized means of the intensity (with Vehicle set at 100%) from a single animal (6 slices /animal). Statistical analysis was performed by one-way ANOVA with post hoc Bonferroni correction (*, p<0.0001 compared to Vehicle, #, p<0.0001 compared to Rotenone).

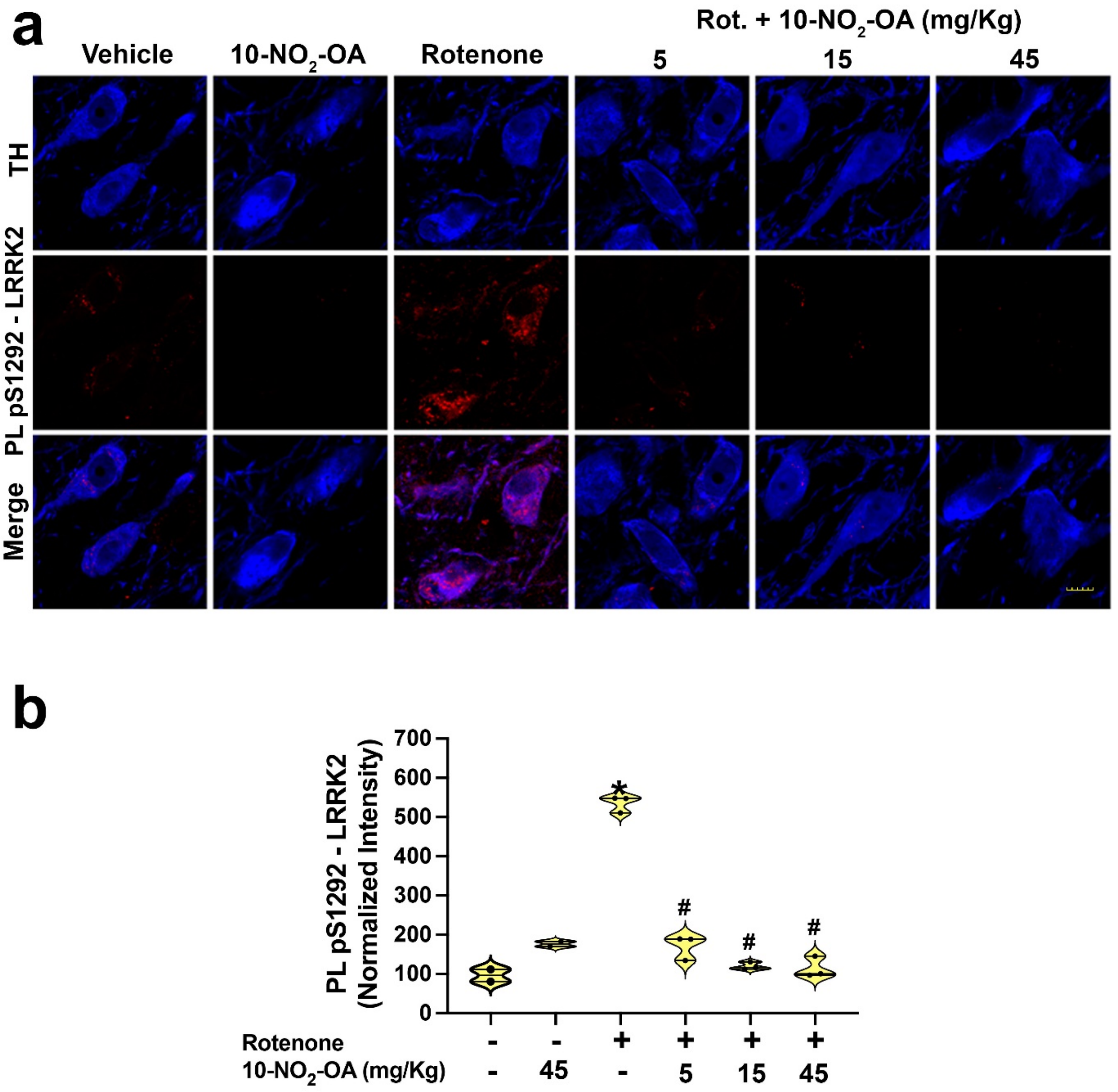

Supplementary Figure 7. 10-NO₂-OA prevented rotenone-induced LRRK2 activation in SNpc. (a) PL pS1292-LRRK2 (red) in dopaminergic neurons (TH, blue) in the SNpc of cohort 1 animals (Scale bar: 20μm). (b) Quantifications of the fluorescence signal. Symbols represent the normalized means of the intensity (with Vehicle set at 100%) from a single animal (6 slices /animal). Statistical analysis was performed by one-way ANOVA with post hoc Bonferroni correction (*, $p<0.0001$ compared to Vehicle, #, $p<0.0001$ compared to Rotenone).

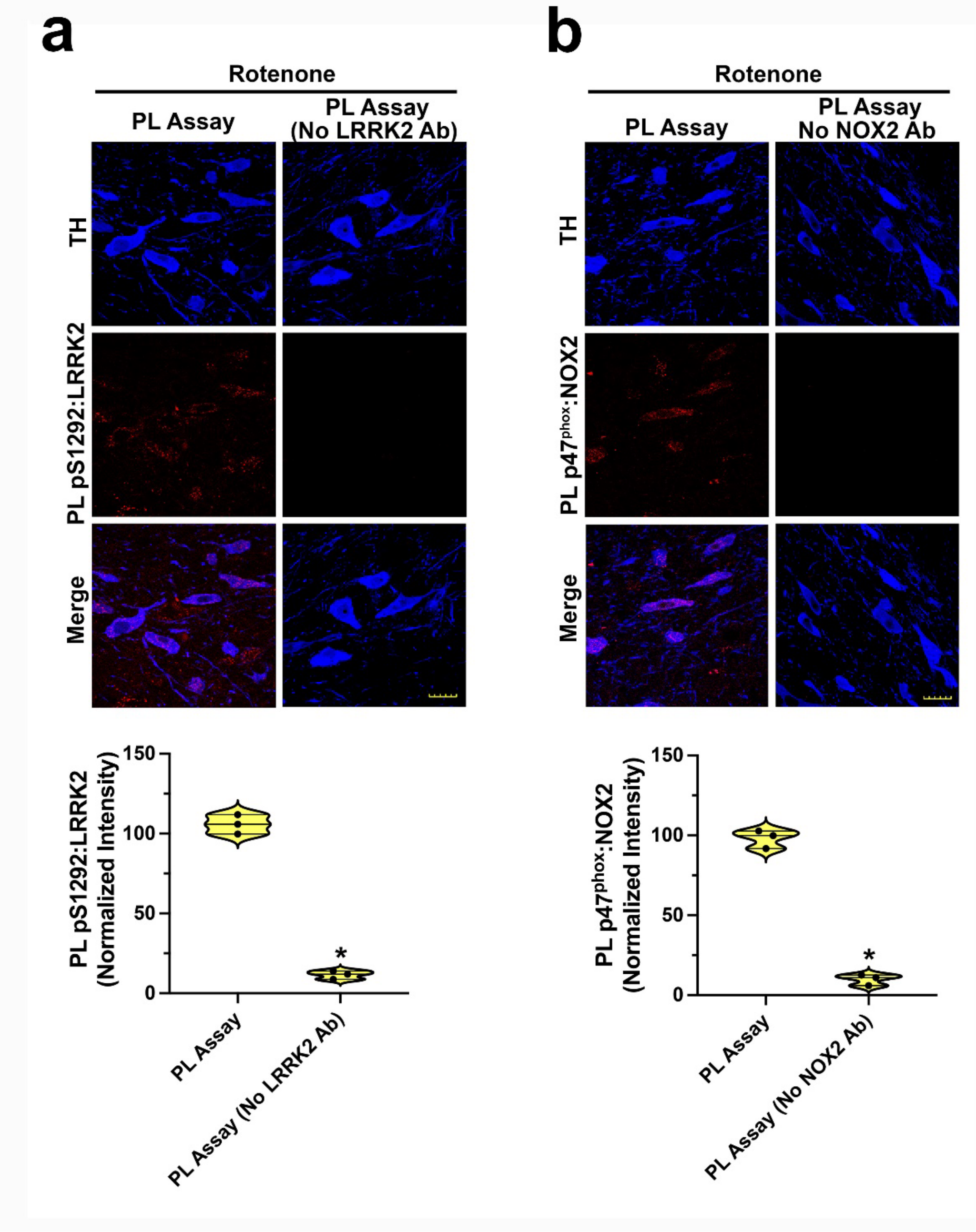

Supplementary Figure 8. Validation of PL assays for LRRK2 and NOX2 by antibody depletion. (a, upper panel) PL pS1292:LRRK2 signal (red) disappears in dopaminergic neurons (TH, blue) not exposed to LRRK2 (N241A) Ab (Scale bar: 30μm). (a, lower graph) Quantification of the PL-related fluorescence signal. (b, upper panel) PL $p47^{phox}$: NOX2 signal (red) disappears in dopaminergic neurons (TH, blue) not exposed to NOX2 ($gp91^{phox}$) Ab (Scale bar: 30μm). (b, lower graph) Quantification of the PL-related fluorescence signal. Symbols represent the normalized means of the intensity (with rotenone set at 100%) from a single animal (6 slices/animal). Statistical analysis was performed by unpaired t-test (*, p<0.0001).

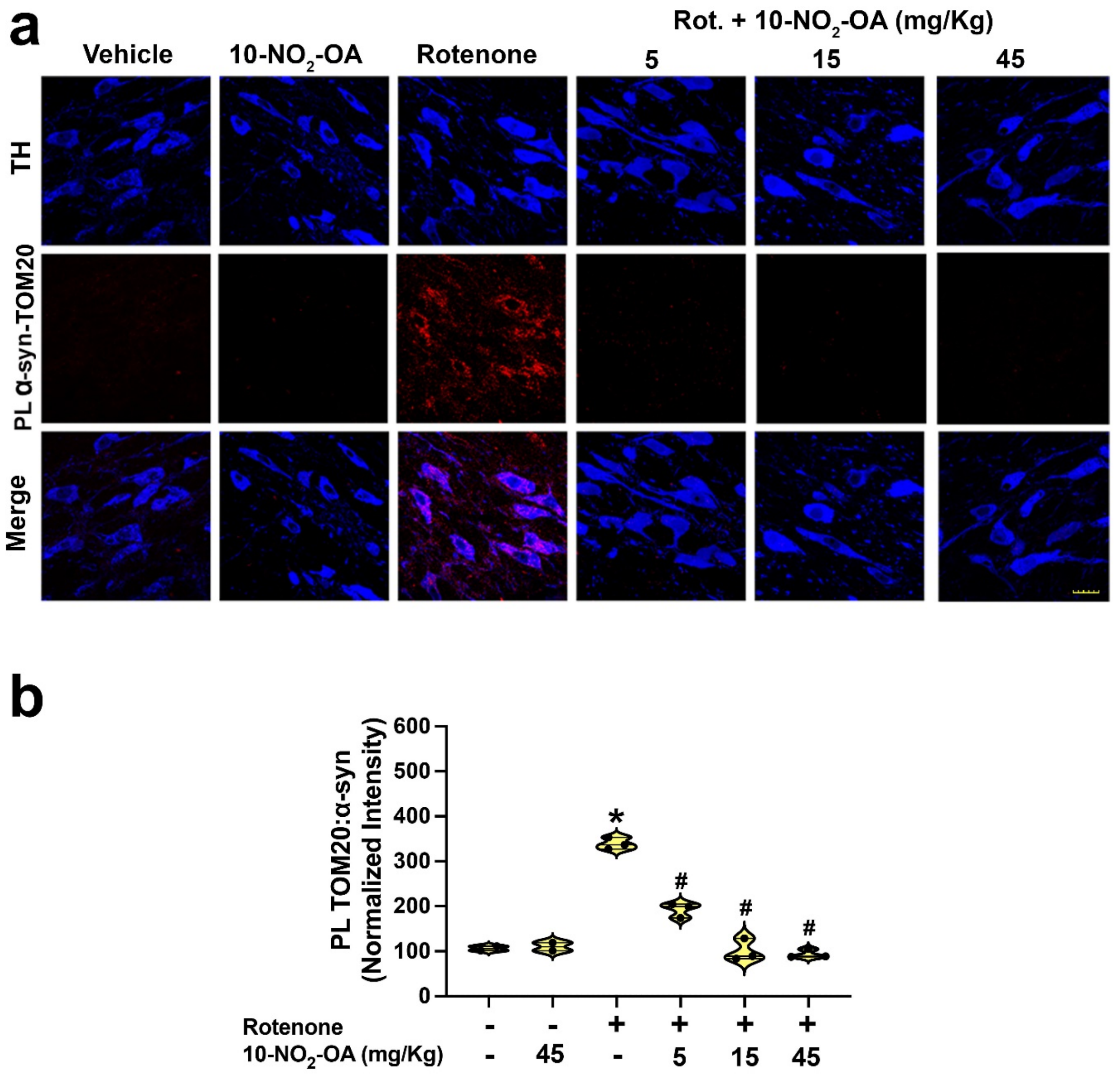

Supplementary Figure 9. 10-NO2-OA inhibits α-synuclein-mediated mitochondrial import impairment in the subacute PD model of rotenone. (a) Confocal microscopy of PL α-synuclein:TOM20 (red) in dopaminergic neurons (TH, blue) in the SNpc of rats (cohort 1 - Scale bar: 30μm). (b) Quantification of the fluorescence signal. Symbols represent the normalized means of the intensity (with Vehicle set at 100%) from a single animal (6 slices /animal). Statistical analysis was performed by one-way ANOVA with post

hoc Bonferroni correction (*, p<0.0001 compared to Vehicle, #, p<0.0001 compared to Rotenone).

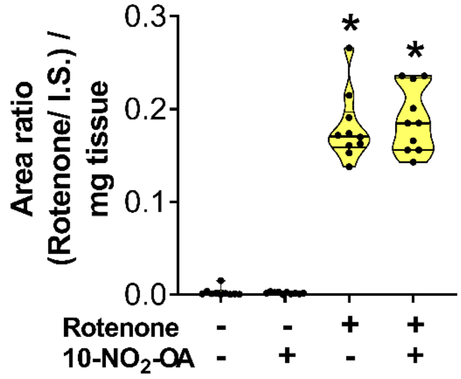

Supplementary Figure 10. Analysis of rotenone in whole rat brain (cohort 2). HPLC-MS-MS analysis of rotenone levels. Statistical analysis was performed by one-way ANOVA with post hoc Bonferroni correction (*, p<0.0001 compared to Vehicle, and 10-NO_2-OA).
